# The Potential Prognostic Role of Oligosaccharide-Binding Fold-Containing Protein 2A (OBFC2A) in Triple-Negative Breast Cancer

**DOI:** 10.3389/fonc.2021.751430

**Published:** 2021-11-15

**Authors:** Qianxue Wu, Xin Tang, Wenming Zhu, Qing Li, Xiang Zhang, Hongyuan Li

**Affiliations:** ^1^ Department of the Endocrine and Breast Surgery, The First Affiliated Hospital of Chongqing Medical University, Chongqing Medical University, Chongqing, China; ^2^ Department of Oncology, Chongqing University Cancer Hospital, Chongqing, China

**Keywords:** OBFC2A, triple-negative breast cancer, prognosis, overall survival, molecular biology

## Abstract

**Background:**

Patients with triple-negative breast cancer (TNBC) have poor overall survival. The present study aimed to investigate the potential prognostics of TNBC by analyzing breast cancer proteomic and transcriptomic datasets.

**Methods:**

Candidate proteins selected from CPTAC (the National Cancer Institute’s Clinical Proteomic Tumor Analysis Consortium) were validated using datasets from METABRIC (Molecular Taxonomy of Breast Cancer International Consortium). Kaplan-Meier analysis and ROC (receiver operating characteristic) curve analysis were performed to explore the prognosis of candidate genes. GO (Gene Ontology) and KEGG (Kyoto Encyclopedia of Genes and Genomes) enrichment analysis were performed on the suspected candidate genes. Single-cell RNA-seq (scRNA-seq) data from GSE118389 were used to analyze the cell clusters in which OBFC2A (Oligosaccharide-Binding Fold-Containing Protein 2A) was mainly distributed. TIMER (Tumor Immune Estimation Resource) was used to verify the correlation between OBFC2A expression and immune infiltration. Clone formation assays and wound healing assays were used to detect the role of OBFC2A expression on the proliferation, invasion, and migration of breast cancer cells. Flow cytometry was used to analyze the effects of silencing OBFC2A on breast cancer cell cycle and apoptosis.

**Results:**

Six candidate proteins were found to be differentially expressed in non-TNBC and TNBC groups from CPTAC. However, only OBFC2A was identified as an independently poor prognostic gene marker in METABRIC (HR=3.658, 1.881-7.114). And OBFC2A was associated with immune functions in breast cancer. Biological functional experiments showed that OBFC2A might promote the proliferation and migration of breast cancer cells. The inhibition of OBFC2A expression blocked the cell cycle in G1 phase and inhibited the transformation from G1 phase to S phase. Finally, downregulation of OBFC2A also increased the total apoptosis rate of cells.

**Conclusion:**

On this basis, OBFC2A may be a potential prognostic biomarker for TNBC.

## Introduction

Breast cancer has the highest incidence of malignant tumors among women worldwide, posing a serious threat to their health (1). The classification system that can explain the heterogeneity of breast cancer divides breast cancer into the following subtypes: luminal A-like subtype, luminal B-like subtype, HER2 subtype, non-luminal or luminal, and basal-like subtype (2). More than 90% of basal-like breast cancers represent TNBC (triple-negative breast cancer), and basal-like breast cancer is the most common type of TNBC (3). Existing studies have shown that the specific mechanism of TNBC is not clear. Biologically, TNBC tumors tend to be more aggressive and larger, with higher oncology grades and lymph node metastasis. Due to the lack of clear molecular targets, the drug treatment of TNBC depends on chemotherapy. Compared with other breast cancer subtypes, TNBC patients have a higher long-term recurrence rate and poor prognosis (4). Therefore, new prognostic factors and therapeutic targets for TNBC need to be explored.

In tumor research, genes that promote the occurrence and development of cancer were often screened in gene expression profiles (5). In recent years, due to the rise of proteomics, screened biomarkers were more in line with clinical characteristics (6). Gillette et al. (7) performed a comprehensive proteomic characterization to explore the biology of lung adenocarcinoma and identify new therapeutic opportunities. In addition, Clark et al. have shown that CPTAC had performed a comprehensive proteogenomic characterization to elucidate the impact of genomic alterations driving phenotypic changes and outline the mechanisms of clear cell renal cell carcinoma pathobiology (8). In general, a comprehensive analysis of the proteome and transcriptome has the potential to reveal the characteristics of new diseases (9).

In this study, we hypothesized that certain genes play a role in the occurrence and development of TNBC and thus can be used to predict the prognosis of patients with TNBC. We herein investigated the datasets of breast cancer from CPTAC to compare the proteomics datasets of TNBC and non-TNBC groups to explore potential prognostic genes for TNBC. Furthermore, we used the METABRIC dataset for verification. Then, OBFC2A promoted TNBC cell proliferation, migration, and the transformation from G1 phase to S phase, inhibited cell apoptosis. By combining proteomics and transcriptomics to discover promising cancer biomarkers, it was concluded that OBFC2A may be indicative of unfavorable prognosis in TNBC.

## Material and Methods

### Patient Data Acquisition

Breast cancer proteomics datasets (TCGA Cancer Proteome Study of Breast Tissue) and clinical data were downloaded from the CPTAC website (https://proteomics.cancer.gov/programs/cptac). A total of 105 TCGA breast cancer samples were analyzed using iTRAQ (isobaric tags for relative and absolute quantification) protein quantification methods. The samples included four breast cancer subtypes (luminal A, luminal B, basal-like, and HER2-enriched). After excluding patients with incomplete clinical pathological data, this study enrolled 101 patients for subsequent analysis. The scRNA-seq (single-cell RNA-seq) data (1,534 cells in six fresh triple-negative breast cancer tumors) of GSE118389 (10) were also downloaded from GEO (Gene Expression Omnibus) (https://www.ncbi.nlm.nih.gov/geo/). Gene expression and clinical data of 1,904 breast cancer patients from the METABRIC (Molecular Taxonomy of Breast Cancer International Consortium) breast studies were downloaded from the cBioPortal (https://www.cbioportal.org/).

### Differentially Expressed Protein Identification

The batch correction of the CPTAC datasets was performed using ‘Limma’ package (version 3.44.3) and ‘impute’ package version 1.62.0 of R language (version 4.0.0). The R language was used to analyze differentially expressed proteins in the non-TNBC (n=77) and TNBC (n=24) groups. Differentially expressed protein cutoff criteria were a FDR (false discovery rate) corrected p-value<0.01, and logFC > 0.

### GO (Gene Ontology) and KEGG (Kyoto Encyclopedia of Genes and Genomes) Pathway Enrichment Analysis

The ‘clusterProfiler’ package (version 3.16.0), ‘org.Hs.eg.db’ package (version 3.11.4), ‘enrichplot’ package (version 1.8.1), ‘digest’ package (version 0.6.25), ‘GOplot’ package (version 1.0.2) and ‘ggplot2’ package (version 3.3.1) of R language were used to perform GO analysis to expound the BPs (biological processes), MFs (molecular functions), CCs (cellular components) and KEGG analysis.

### Survival Analysis

The correlation between OS (overall survival) and candidate genes or proteins was identified through Kaplan-Meier survival analysis using ‘survminer’ package of R language. A total of 101 breast cancer patients and clinical data from CPTAC, and 1904 breast cancer patients and clinical data from METABRIC, were selected for survival analysis.

### Receiver Operating Characteristic Analysis

The ‘survivalROC’ package (version 1.0.3) of R language was used to calculate the ROC (receiver operating characteristic) curve of candidate proteins, the selecting criteria being AUC > 0.7. The candidate genes were predicted using the ROC curve of TNBC with the ‘pROC’ package (version 1.16.2) of R language.

### Analysis of scRNA-Seq Data

To identify the cells that significantly express OBFC2A, scRNA-seq data of 1,534 cells in six fresh triple-negative breast cancer tumors from GSE118389 were analyzed using the Serat package and the SingleR package in the R language. The specific steps of the scRNA-seq data analysis were carried out as described earlier (11).

### Correlation Analysis of Immune Cell Infiltration

The Pearson correlation coefficient was employed to evaluate the correlation between OBFC2A mRNA expression levels and the level of immune cell infiltration. TIMER gene modules were utilized to investigate OBFC2A expression in diverse tumors and the relationship between OBFC2A expression and the abundance of immune infiltrates. The infiltration data of B cells, CD4 + T cells, CD8 + T cells, dendritic cells, macrophages, and neutrophils can be downloaded from the TIMER database (https://cistrome.shinyapps.io/timer/).

### Cell Culture

The breast cancer cell lines MCF10A, BT549, MDA-MB-231, MCF7, SK-BR-3, YCCB1, ZR75-1, and T47D were obtained from the Cell Bank of the Chinese Academy of Sciences (Shanghai, China). MCF10A culture was performed as described by Lomoriello et al. (12), and the rest of the cell lines were cultured in 90% RPMI-1640 (Gibco, USA) + 10% fetal bovine serum (Biological Industries, Israel) at 37°C in an atmosphere of 5% CO_2_.

### Western Blot

All cells were lysed in RIPA buffer to isolate total proteins. After extracting proteins and measuring the concentration with the BCA protein kit (Beyotime, China), equal amounts of denatured proteins (40 µg) were used for western blotting as previously described (13). The same amount of denatured protein (40 μg) and 10% SDS-PAGE were used for electrophoresis and then transferred to the PVDF membrane. After blocking in 5% non-fat milk for 1h at RT (room temperature), the membrane was incubated with the primary antibody overnight at 4˚C, and then incubated with the second antibody (anti-rabbit IgG or anti-mouse IgG) at RT for 1 h. The Fusion FX7 Spectra multifunction imaging system was used to detect bands. The following primary antibodies were used: OBFC2A (16719-1-AP, Proteintech Group, USA) and GAPDH (GB11002, Servicebio, Hubei, China).

### Lentiviral Transfection

The shRNA targeting OBFC2A (sh-OBFC2A) was constructed by TSINGKEBIO (Tsingkebio Co., Ltd, Beijing, China). The lentivirus shRNA sequence was as follows: 5-GATCGTGCAAAGTAGCAGATA-3′. The second generation lentivirus packaging system was used for generate lentivirus. According to the instructions, Lipofectamine 2000 was used to transfect the lentiviral vector (20 μg, PxpAx2: PMD2g: pLVX = 3: 1: 4) into HEK293T cells at 37°C for 48h, and the lentivirus was generated by transfecting the packaging plasmid with calcium phosphate. When MDA-MB-231 cell density reached 30-50%, the cells were infected with lentivirus at 37°C according to the protocol provided by the manufacturer. At 48 h after transfection, the stable cell lines were selected by using puromycin for 48-72 h. Stable cell lines were selected using 2 µg/ml puromycin. Stable cell lines were used in subsequent cell biology experiments.

### Clone Formation Assay

The colony formation assay was performed as previously described (14). The cells of each group were inoculated into 12-well plates with 1000 cells in each well and three multiple holes in each group, which were cultured in an incubator for one week. Cells were then fixed with 4% paraformaldehyde for 15 min and stained with crystal violet (C0121, Beyotime) for 15 min.

### Wound Healing Assay

Wound healing assays were performed as described previously (15). The cells were inoculated into 6-well plates to form a confluent monolayer at 37°C for 24 h. One wound per well was conducted with a 10-ul tip and washed twice with PBS. The cells were then replaced with serum-free medium, and live cell imaging was performed at 0 and 24 h.

### Cell Cycle

Cells were washed twice with PBS and fixed in 70% cold ethanol at 4°C for 30 min. The cells were rewashed with PBS and incubated with 100 μl RNase A (0.1 mg/ml) and 2 μl PI (propidium iodide) for 30 min at 37°C in the dark. Cell cycle distribution was analyzed *via* flow cytometry (BD CellQuest Pro, version 5.1) using a FACSCalibur (BD Biosciences).

### Apoptosis

Approximately 1 × 10^6^ cells in each group were washed with PBS and suspended in 100 μL of binding buffer. After incubation with Annexin V-FITC (20 μg/ml) and PI (50 μg/ml) (both Sigma-Aldrich; Merck KGaA) at RT for 30 min, apoptosis was detected by FACSCalibur flow cytometry (BD Biosciences). Total cell apoptosis was calculated as the percentage of early + late apoptotic cells.

### Statistical Analysis

The analysis was performed using R language and SPSS software (version 19.0). Student’s t-tests were used to conduct differential comparisons of two groups. Kruskal-Wallis’s test was used to compare multiple independent samples. P value <0.05 was considered to be statistically significant.

## Results

### OBFC2A Is Upregulated in High-Grade Breast Cancer: A Potential Marker for TNBC

**Figure 1:**
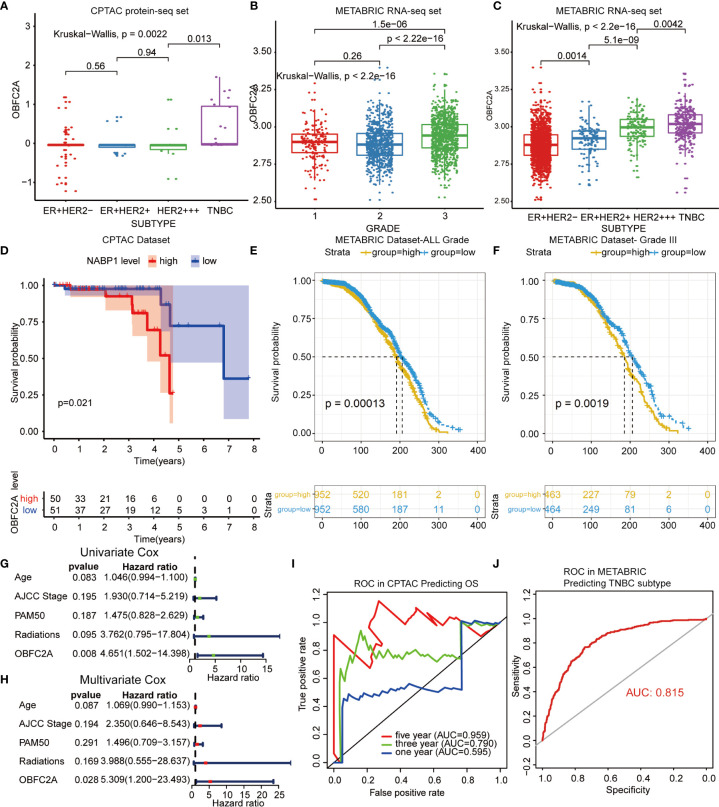
OBFC2A is an independent prognostic factor for patients with breast cancer. **(A–C)** OBEC2A mRNA expression is higher in high-grade samples and TNBC subtype. **(D)** OBFC2A was negatively associated with OS of breast cancer (P < 0.05). **(E, F)** OBFC2A mRNA expression was negative associated with OS of all grade breast cancer(P < 0.05). **(G, H)** Univariate Cox and Multivariate Cox regression analyses were performed in CPTAC. **(I, J)** ROC curves of OBFC2A expression to predict OS of three year (0.790) and five year (0.959) in CPTAC and TNBC subtype (0.815) in METABRIC.

A total of 1565 differentially expressed proteins were identified from CPTAC (**Table S1**). **Figure S1** showed the PPI (protein-protein interaction network) of differentially expressed genes. Finally, six candidate proteins (DLAT, ELAVL2, KARS, OBFC2A, RAVER2, SSBP1) were selected by survival analysis filtering from CPTAC proteomics datasets (**Figures S2**, **S3**). Only OBFC2A was validated in METABRIC dataset. As shown in **Figure 1B**, OBFC2A mRNA expression was higher in grade III (p < 0.05). To explore the expression distribution of OBFC2A, the expression of OBFC2A in different subtypes was performed. The results showed that the expression levels of OBFC2A were higher in the TNBC group than in the other subtypes, in both CPTAC and METABRIC (**Figures 1A, C**). To further confirm this finding, the ROC was evaluated for OBFC2A expression and TNBC subtype of all breast cancers. The results showed that AUC (the areas under the curve) was 81.5% (TNBC) in the METABRIC (**Figure 1J**).

### OBFC2A Is an Independent Prognostic Factor for Patients With Breast Cancer

To explore the relationship between OBFC2A expression and overall survival, the overall survival of each group was analyzed. The results showed that the OS of patients with higher OBFC2A expression was shorter than that of patients with lower OBFC2A expression in CPTAC and METABRIC (**Figures 1D–F**; p < 0.05). To further confirm this finding, the ROC was evaluated for OBFC2A expression and OS of all grade breast cancers. The results showed that the AUC were 79% (three years) and 95.9% (five years) in the CPTAC (**Figure 1I**). To explore OBFC2A was an independent prognostic factor, Univariate (**Figure 1G**) and Multivariate (**Figure 1H**) Cox regression analyses were performed in CPTAC and METABRIC. Multivariate Cox regression analysis showed that OBFC2A was independently associated with OS in CPTAC and METABRIC, respectively (HR = 5.309, 1.200-23.493, P=0.028; HR = 3.658, 1.881-7.114, p < 0.0001) (**Figure 1H** and **Table 1**). 

**Table 1:** Univariate and multivariate Cox in METABRIC RNA-seq set.

Clinical factor	Univariate				Multivariate			
	HR	CI95.low	CI95.high	P.value	HR	CI95.low	CI95.High	P.value
Age	0.991	0.985	0.997	0.003547	1.002	0.995	1.009	0.613026
size	1.193	0.78	1.825	0.416574	1.061	0.69	1.633	0.786536
LN Metastasis	1.158	1.002	1.339	0.046674	0.839	0.701	1.003	0.054062
radiotherapy	1.408	1.21	1.638	1.00E-05	1.407	1.201	1.648	2.40E-05
chemotherapy	1.779	1.503	2.106	0	1.989	1.557	2.541	0
grade	1.006	0.872	1.159	0.939048	0.837	0.715	0.98	0.026982
Subtype	1.181	0.98	1.423	0.079825	0.787	0.626	0.988	0.03943
OBFC2A	3.612	2.009	6.493	1.80E-05	3.658	1.881	7.114	1.33E-04

### OBFC2A May Be Associated With Immune Functions in Breast Cancer

**Figure 2:**
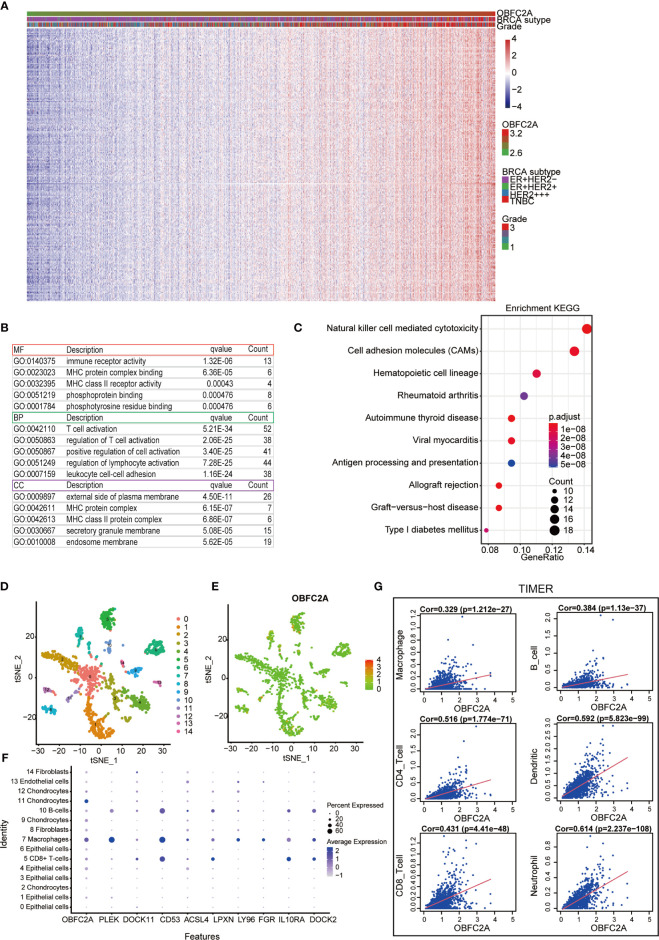
Correlation between OBFC2A and immune function. **(A)** 236 genes positively related with OBFC2A expression (R > 0.5). **(B, C)** The GO and KEGG analysis results show that OBFC2A expression is related to immune function of breast cancer. **(D–F)** Scatter plots and Bubble plots showing the distribution of OBFC2A expression in different cell clusters, annotated according to the analysis of the SingleR package; **(G)** The correlation coefficients between the mRNA expression of OBFC2A and macrophages, B cells, CD8 + T cells, CD4 + T cells, DC cells and neutrophils, and OBFC2A were 0.329, 0.384, 0.431, 0.516, 0.592, 0.614, respectively.

To explore the biological function associated with OBFC2A expression in breast cancer, Pearson correlation analysis between OBFC2A expression and other genes in whole-genome profiling of 1904 patients in METABRIC was performed. The results showed that 236 genes were positively correlated with OBFC2A expression (R > 0.5) (**Figure 2A**). GO and KEGG pathway enrichment analyses of the above genes were performed. The results were listed in **Figures 2B, C**, wherein 3 MFs, 3 BPs, and 3 CCs for 236 genes were observed including immune receptor activity, MHC protein complex binding, MHC class II receptor; T cell activation, regulation of T cell activation, positive regulation of cell activation; external side of plasma membrane, MHC protein complex, MHC class II protein complex. As for KEGG analysis, natural killer cell mediated cytotoxicity, cell adhesion molecules (CAMs), and hematopoietic cell lineage were included. In the t-SNE analysis, 14 cell clusters were found, and the scatter plot showed that OBFC2A was not evenly expressed among the cell clusters (**Figures 2D, E**). Moreover, the scatter diagram and bubble diagram showed that OBFC2A and its related genes were specifically expressed in macrophages (**Figure 2F**). Since OBFC2A was possibly related to tumor immunity, TIMER database was used to analyze the correlation between OBFC2A and immune cell subtype infiltration. As shown in **Figure 2G**, the correlation coefficients between macrophages, B cells, CD8 + T cells, CD4 + T cells, DCs, and neutrophils, and OBFC2A were 0.329, 0.384, 0.431, 0.516, 0.592, and 0.614, respectively (P < 0.001).

### OBFC2A Regulates Breast Cancer Cell Proliferation and Metastasis

**Figure 3:**
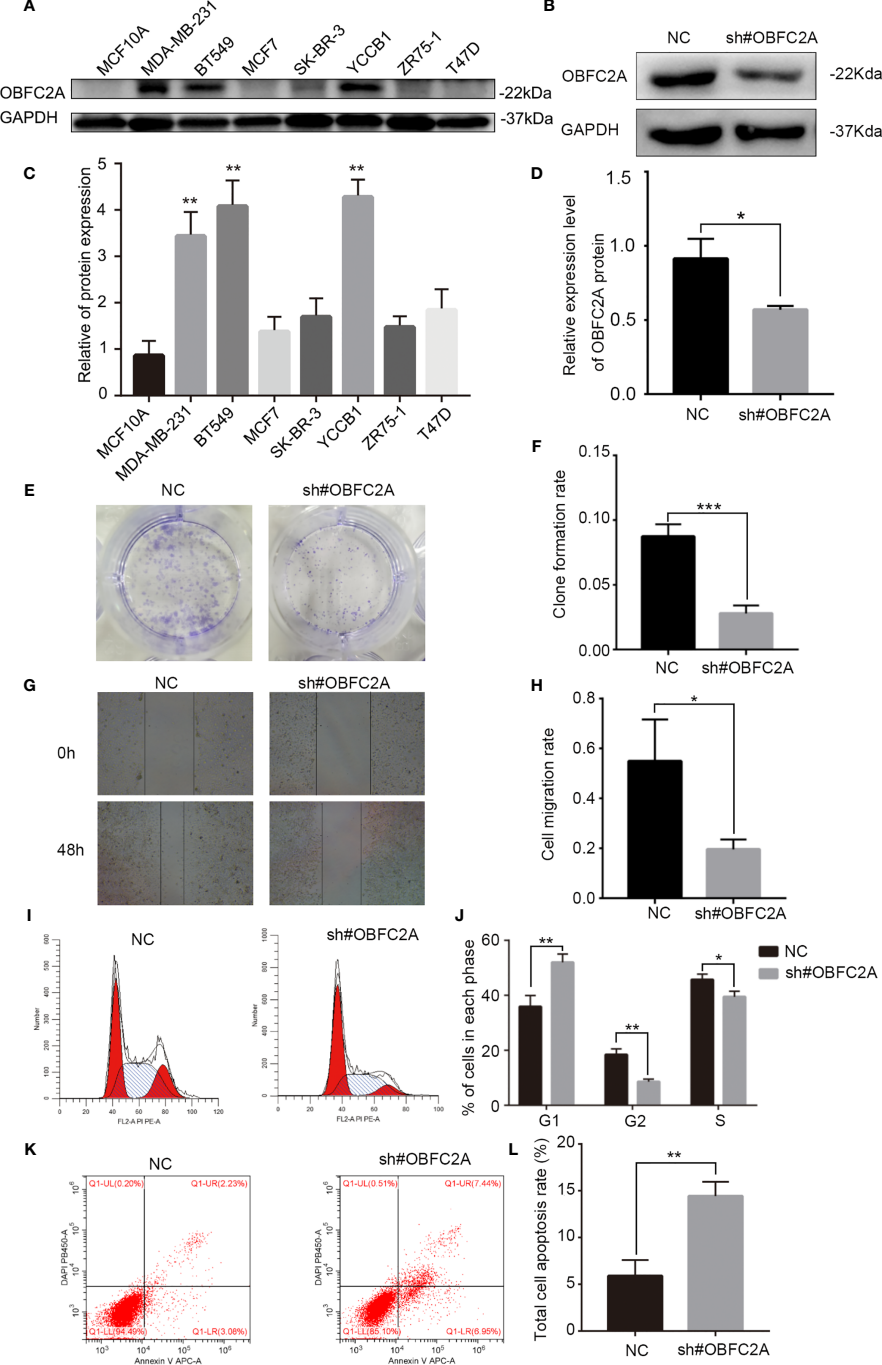
OBFC2A plays a role in promoting the development of breast cancer. **(A, C)** OBFC2A protein expression is also higher in TNBC subtype and cell lines of TNBC (MDA-MB-231, BT549, YCCB1). **(B, D)** Western blot analysis confirmed the silencing efficacy; **(E, F)** Colony formation assay with cells transfected with NC or OBFC2AshRNA (12-well plate); **(G, H)** OBFC2A-silenced MDA-MB-231 cell exhibited significantly decreased cell motility. Flow cytometry was used to analyze the effects of silencing OBFC2A on cell cycle **(I, J)** and apoptosis **(K, L)**. Data are presented as mean ± SD, n=3, *p < 0.05, **P < 0.01, ***p < 0.001.

It was further confirmed that OBFC2A was highly expressed in TNBC cell lines (BT549, MDA-MB-231, and YCCB1) by verifying the protein expression in all breast cancer cell lines (**Figures 3A, C**, P<0.01). To determine the role of OBFC2A in the development of breast cancer, the function of OBFC2A in breast cancer cells was investigated. A lentiviral transfection system was used to knockdown the protein expression of OBFC2A in the MDA-MB-231 cell line (**Figures 3B, D**). Clone formation assays and wound healing assays showed that knockdown of OBFC2A inhibited the proliferation and migration of TNBC cells (**Figures 3E–H**). The results of flow cytometry showed that silencing OBFC2A promoted apoptosis and blocked cell cycle in G1 phase (**Figures 3I–L**).

## Discussion

In this study, bioinformatics methods were used to screen and identify OBFC2A from the database as a possible risk prognostic factor for TNBC. Biological function experiments indicated that OBFC2A might promote the occurrence and development of TNBC. TNBCs are complex and heterogeneous neoplasms that are often characterized by aggressive behavior and limited therapeutic possibilities (16). The complexity of breast cancer treatment is attributed to the heterogeneity of breast cancer (17). Treatment decisions depend on three markers: the expression of estrogen and progesterone receptors, and the expression status of HER2 (18). Compared with other subtypes of breast cancer, TNBC has strong heterogeneity, tends to have a higher tumor grade, is accompanied by lymph node metastasis, and lacks the expression of the above three markers; therefore, there are no specialized targeted drugs, and the prognosis of patients with TNBC is usually relatively poor (19, 20).

In recent years, bioinformatics has been widely used to identify various cancer prognosis-related genes (21). Zheng et al. identified potential prognostic biomarkers in cancer through joint RNA-seq analysis from TCGA and proteomic analysis from CPTAC (22, 23). Peng et al. integrated analysis of TCGA genomic data and CPTAC proteomic data to validate the important contribution of A-to-I RNA editing to protein diversity in cancer (24). When developing new clinical markers related to breast cancer, the common method is to compare the difference in gene expression between patients with TNBC and non-TNBC patients (25). Here, we found that the expression of OBFC2A in the TNBC group was higher than that in the non-TNBC group, and that OBFC2A was an independent risk predictive factor in breast cancer. Vernin et al. found that OBFC2A was inactivated by oncomiRNA, thereby promoting CD4+T cell proliferation and genetic instability (26). Our research also found that OBFC2A-related genes were enriched in immune-related pathways. OBFC2A was found to be expressed in macrophages clusters in single-cell sequencing analysis. TIMER immune cell infiltration analysis revealed that OBFC2A expression was significantly correlated with immune cell infiltration. These results suggested that the interaction between OBFC2A and tumor-associated macrophages may drive the development of breast cancer. Tumor-derived UBR5 was essential for cancer progression by promoting tumor-associated macrophage recruitment and activation *via* key chemokines and cytokines (27). It also showed that targeting tumor-associated macrophage-related gene (OBFC2A) may have broader implications for improving the effectiveness of cancer therapy.

Collectively, these results suggest a new role for OBFC2A in breast cancer. OBFC2A, a single-stranded DNA binding protein, may be involved in the regulation of cancer cell DNA damage and repair, thereby inhibiting cancer cell death. Kang et al. first reported that OBFC2A was a single-stranded nucleic acid-binding protein, which might be associated with DNA recombination or repair of thymocytes (28). Studies have also shown that OBFC2A was indispensable for embryogenesis and adult tissue homeostasis (including thymopoiesis, spleen development, male fertility, and DNA repair in mice) (29). Moreover, Won et al. showed for the first time that OBFC2A was involved in human diseases and variant acute promyelocytic leukemia (30). OBFC2A also triggered the repair mechanism to maintain genome stability and targeted Smad3 regulated cyclin D1 that influences cell cycle arrest (31, 32). Homologous recombination repair of DNA double strand breaks could induce breast cancer cells to develop chemoresistance (33). Our results also showed that downregulation of OBFC2A expression in TNBC cells inhibited breast cancer cell proliferation and migration, promoted cell apoptosis, blocked cell cycle in G1 phase, indicating its role in the occurrence and development of TNBC.

The innovative point of this study is to combine the proteome and transcriptome datasets for gene screening. Proteomics can better reflect the role of genes in cancer biology. The limitations of this study are as follows. Firstly, the correlation between OBFC2A expression and immune function has not been further verified by experiments. Furthermore, the role of OBFC2A *in vivo* has not been verified by our study. OBFC2A may be associated with DNA damage repair or immunity, so it is of great clinical significance to explore the involvement of OBFC2A in TNBC chemoresistance *via* immune evasion or homologous recombination repair in the future research.

## Conclusion

In our study, OBFC2A was upregulated in TNBC, and OBFC2A was negatively correlated with OS. In addition, OBFC2A-related genes may be related to immune function. Finally, the inhibition of OBFC2A attenuated the proliferation and migration, promoted cell apoptosis, blocked cell cycle in G1 phase of TNBC cells. Viewed in total, OBFC2A may be regarded as a potential prognostic factor for TNBC.

## Data Availability Statement

Data used in this publication were generated by the Clinical Proteomic Tumor Analysis Consortium (CPTAC, NCI/NIH). Breast cancer proteomics datasets can be found in CPTAC (https://proteomics.cancer.gov/programs/cptac). And METABRIC datasets also can be found in cBioPortal (https://www.cbioportal.org/).

## Author Contributions

QW: Conceptualization (lead); Data Curation (lead); Methodology (lead); Formal analysis (lead); Writing-original draft (lead). XT: Data Curation (equal); Formal analysis (supporting). QL: Data Curation (equal); Formal analysis (supporting). WZ: Data Curation (equal); Formal analysis (supporting). XZ: Supervision (equal); Writing-review and editing (equal). HL: Supervision (lead); Writing-review and editing (supporting). All authors contributed to the article and approved the submitted version.

## Funding

This work was supported by the Natural Science Foundation of Chongqing [cstc2020jcyj-msxmX0600].

## Conflict of Interest

The authors declare that the research was conducted in the absence of any commercial or financial relationships that could be construed as a potential conflict of interest.

## Publisher’s Note

All claims expressed in this article are solely those of the authors and do not necessarily represent those of their affiliated organizations, or those of the publisher, the editors and the reviewers. Any product that may be evaluated in this article, or claim that may be made by its manufacturer, is not guaranteed or endorsed by the publisher.

## Supplementary Material

Supplementary Figure 1 The protein-protein interaction network (PPI) of differentially expressed genes.

Supplementary Figure 2 Six proteins were selected from CPTAC proteomics datasets. **(A, B)** The GO and KEGG analysis results show that differentially expressed proteins were related to cancer signaling pathway. **(C-H)** Six proteins (DLAT, ELAVL2, KARS, OBFC2A, RAVER2, SSBP1) were negatively associated with OS of breast cancer (P<0.05).

Supplementary Figure 3 Six proteins were selected from CPTAC proteomics datasets. Six proteins (DLAT, ELAVL2, KARS, OBFC2A, RAVER2, SSBP1) were independently prognostic factors in breast cancer.
