# Supplementary figures and images for: The Potential Prognostic Role of Oligosaccharide-Binding Fold-Containing Protein 2A (OBFC2A) in Triple-Negative Breast Cancer
Source: Front Oncol. 2021 Nov 15;11:751430. doi: 10.3389/fonc.2021.751430 (PMC8634334; doi:10.3389/fonc.2021.751430)

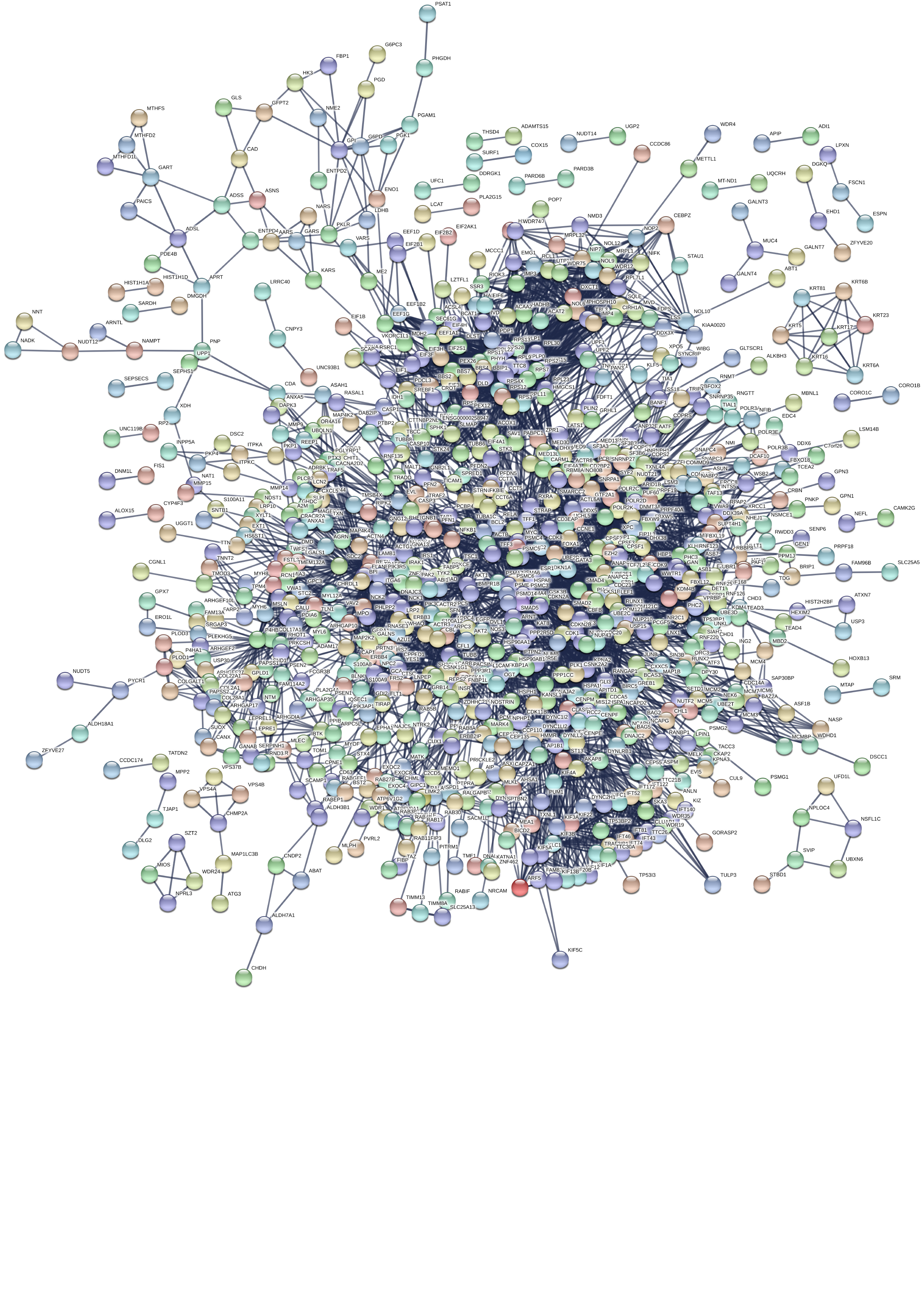

Supplement: Supplementary Figure 1 — The protein-protein interaction network (PPI) of differentially expressed genes. [file Image_1.tif]

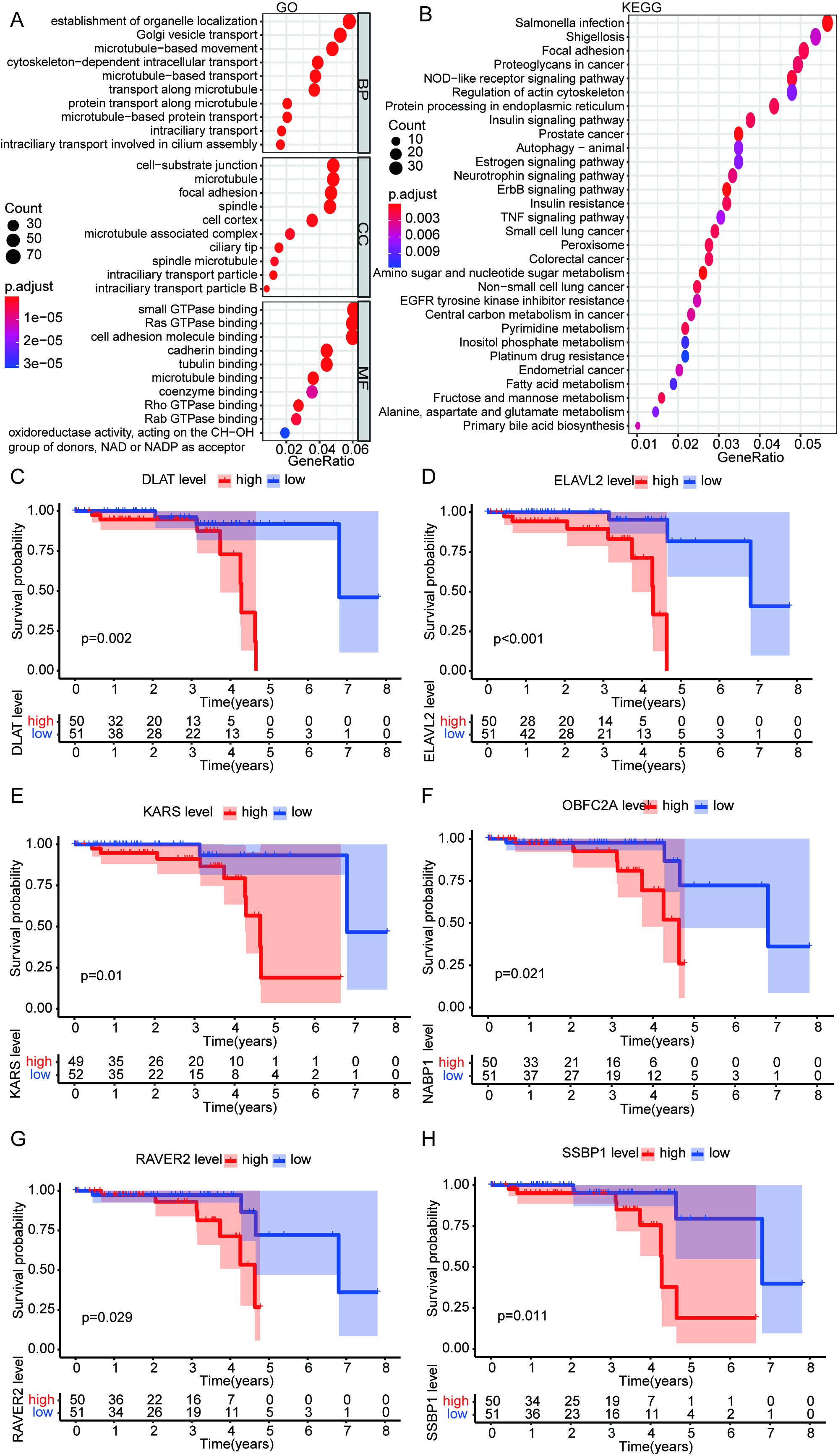

Supplement: Supplementary Figure 2 — Six proteins were selected from CPTAC proteomics datasets. (A, B) The GO and KEGG analysis results show that differentially expressed proteins were related to cancer signaling pathway. (C-H) Six proteins (DLAT, ELAVL2, KARS, OBFC2A, RAVER2, SSBP1) were negatively associated with OS of breast cancer (P<0.05). [file Image_2.tif]

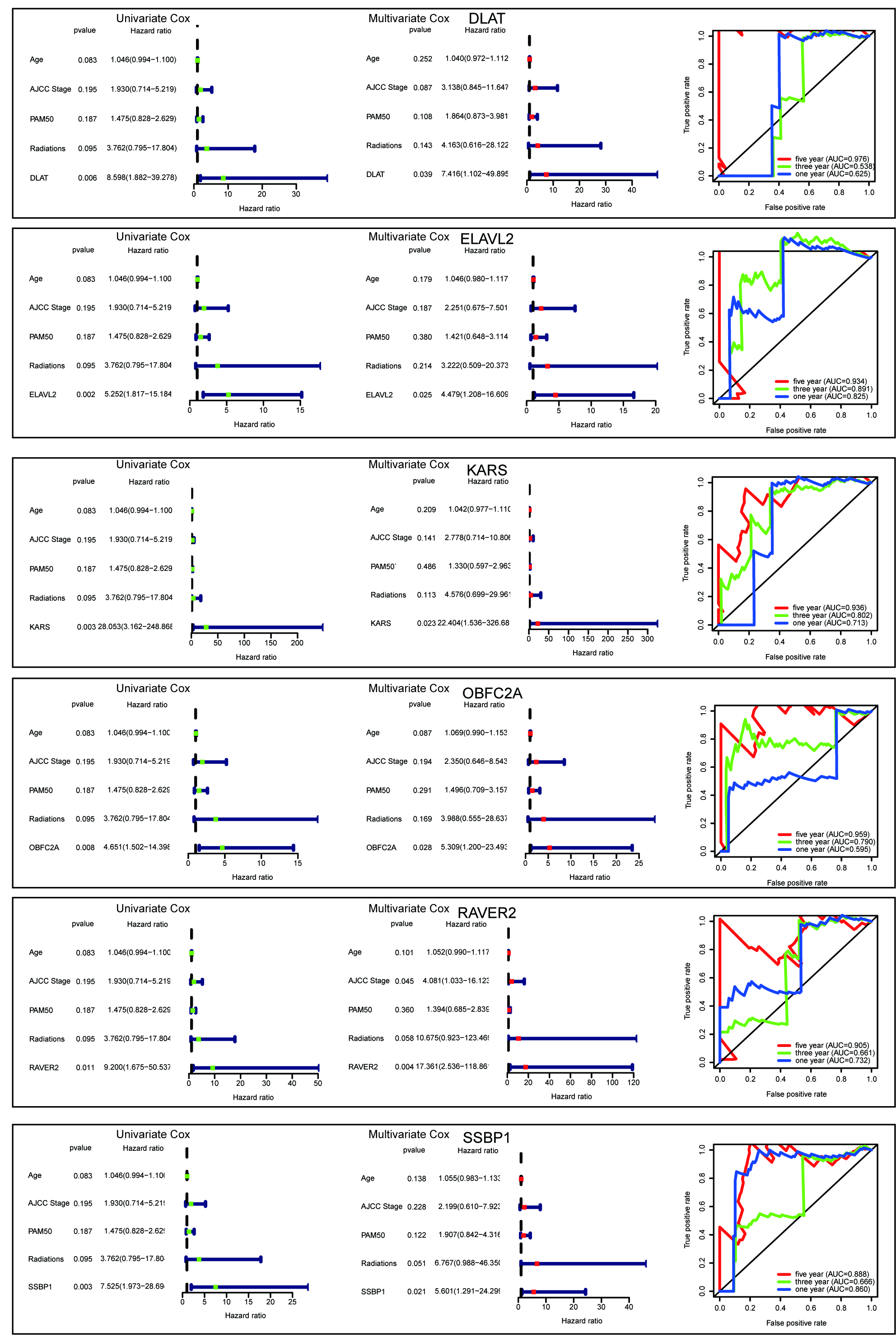

Supplement: Supplementary Figure 3 — Six proteins were selected from CPTAC proteomics datasets. Six proteins (DLAT, ELAVL2, KARS, OBFC2A, RAVER2, SSBP1) were independently prognostic factors in breast cancer. [file Image_3.tif]
