# Supplementary material for: The Potential Prognostic Role of Oligosaccharide-Binding Fold-Containing Protein 2A (OBFC2A) in Triple-Negative Breast Cancer
Source: Front Oncol. 2021 Nov 15;11:751430. doi: 10.3389/fonc.2021.751430 (PMC8634334; doi:10.3389/fonc.2021.751430)
Supplement: Supplementary file 4 [file Table_1.docx]

Table S1 1565 differentially expressed proteins were identified from CPTAC

| gene | Non-TNBC | TNBC | logFC | pValue |
| --- | --- | --- | --- | --- |
| A2M | 0.421963 | -0.07663 | -0.4986 | 0.001566 |
| A2ML1 | 0.505015 | -0.69073 | -1.19574 | 1.69E-05 |
| AAGAB | -0.28465 | 0.206844 | 0.491495 | 1.21E-05 |
| AARS2 | 0.176876 | -0.0261 | -0.20297 | 0.007121 |
| AARSD1 | -0.18064 | 0.005375 | 0.186012 | 0.000712 |
| ABAT | -1.24006 | -0.12697 | 1.113091 | 8.88E-08 |
| ABCB10 | 0.23741 | -0.00519 | -0.2426 | 0.004376 |
| ABCC4 | 0.260231 | -0.03506 | -0.29529 | 0.007177 |
| ABCD3 | -0.95723 | -0.47643 | 0.4808 | 0.005027 |
| ABCF1 | 0.152361 | -0.04542 | -0.19778 | 0.004104 |
| ABHD14B | -0.2392 | 0.16193 | 0.401128 | 0.004871 |
| ABHD4 | -0.13821 | 0.181535 | 0.319741 | 0.00368 |
| ABI1 | 0.239134 | 0.05623 | -0.1829 | 0.000604 |
| ABRACL | 0.385105 | -0.07735 | -0.46245 | 0.003681 |
| ABT1 | 0.393289 | -0.02489 | -0.41818 | 0.001006 |
| ACACA | -0.3189 | 0.075653 | 0.394553 | 0.001016 |
| ACAD9 | 0.187781 | -0.0942 | -0.28198 | 0.00258 |
| ACADSB | -1.10332 | -0.13078 | 0.972531 | 1.98E-07 |
| ACBD3 | -0.05944 | 0.146033 | 0.205477 | 0.00367 |
| ACOT8 | -0.39485 | -0.09647 | 0.298375 | 0.002829 |
| ACOX3 | -0.502 | 0.026135 | 0.528136 | 0.00163 |
| ACSF2 | -0.5267 | 0.037562 | 0.564261 | 0.000191 |
| ACSL4 | 0.502592 | 0.073225 | -0.42937 | 2.77E-05 |
| ACSS3 | -0.60132 | -0.03658 | 0.564738 | 0.001863 |
| ACTN4 | 0.326385 | -0.1132 | -0.43959 | 4.61E-07 |
| ACTR10 | -0.01179 | 0.09503 | 0.106818 | 0.008492 |
| ACTR2 | 0.334613 | 0.096987 | -0.23763 | 0.000269 |
| ACTR5 | 0.078917 | 0.283678 | 0.20476 | 0.000345 |
| ACY1 | -0.49216 | 0.014413 | 0.506575 | 5.98E-05 |
| ADD2 | 0.295509 | -0.16085 | -0.45636 | 0.003214 |
| ADGRE2 | 0.409775 | 0.043545 | -0.36623 | 0.009469 |
| ADGRL2 | 0.435637 | -0.12478 | -0.56042 | 0.002485 |
| ADI1 | 0.241264 | -0.02057 | -0.26184 | 0.007698 |
| ADIPOR1 | -0.50571 | -0.07949 | 0.426212 | 0.002469 |
| ADIPOR2 | -0.4669 | -0.04902 | 0.417882 | 0.002068 |
| ADIRF | -1.20972 | -0.4449 | 0.764817 | 0.00263 |
| ADSL | 0.212823 | -0.0326 | -0.24542 | 0.001516 |
| AES | -0.21565 | 0.075786 | 0.291434 | 0.004902 |
| AFDN | -0.00682 | -0.31093 | -0.30411 | 0.005796 |
| AFTPH | -0.17195 | 0.035172 | 0.207122 | 0.00183 |
| AGA | 0.001151 | 0.369275 | 0.368124 | 0.003411 |
| AGPAT5 | 0.022893 | -0.43373 | -0.45662 | 0.001924 |
| AGR2 | -2.11828 | -0.49242 | 1.625858 | 5.04E-09 |
| AGR3 | -1.58768 | -0.02279 | 1.56489 | 1.61E-11 |
| AGRN | 0.329273 | -0.18635 | -0.51562 | 0.000127 |
| AHCTF1 | 0.13481 | -0.03973 | -0.17454 | 0.005276 |
| AHDC1 | 0.266109 | -0.00953 | -0.27564 | 0.000869 |
| AHNAK | -0.29408 | 0.282901 | 0.57698 | 2.88E-05 |
| AIF1L | 0.562483 | -0.40747 | -0.96995 | 3.07E-06 |
| AKAP5 | -0.73728 | -0.17775 | 0.559522 | 9.25E-05 |
| AKAP8 | 0.151206 | -0.07964 | -0.23084 | 0.00353 |
| AKR1A1 | -0.33028 | -0.03945 | 0.290832 | 0.007391 |
| AKR7A3 | -1.48059 | -0.54155 | 0.939036 | 1.06E-05 |
| AKT1S1 | -0.13001 | 0.084761 | 0.21477 | 0.003258 |
| ALCAM | -0.87429 | -0.14977 | 0.724518 | 0.001593 |
| ALDH16A1 | -0.13472 | 0.074425 | 0.209148 | 0.000912 |
| ALDH1A3 | 0.308302 | -0.52251 | -0.83081 | 3.21E-06 |
| ALDH4A1 | -0.4841 | -0.10218 | 0.381924 | 0.002898 |
| ALDH6A1 | -0.41279 | -0.04525 | 0.367541 | 0.008105 |
| ALDOA | -0.55229 | -0.21351 | 0.338782 | 0.001296 |
| ALLC | -0.57779 | 0.206537 | 0.784323 | 0.000525 |
| ALPL | 0.468965 | -0.13506 | -0.60402 | 0.00218 |
| ALS2 | -0.10438 | 0.114345 | 0.218728 | 0.005302 |
| AMACR | -0.35981 | 0.063699 | 0.423512 | 0.001108 |
| AMDHD2 | -0.01397 | 0.249103 | 0.263074 | 0.004323 |
| AMPD2 | 0.204433 | -0.01679 | -0.22122 | 0.002409 |
| AMY1A | -0.41118 | -1.09493 | -0.68374 | 0.006545 |
| AMZ2 | -0.27081 | 0.074931 | 0.345736 | 0.00251 |
| ANGEL2 | 0.011352 | 0.231433 | 0.220081 | 0.007277 |
| ANKHD1 | -0.10889 | 0.160561 | 0.269453 | 0.000481 |
| ANKRD13D | -0.04741 | 0.197868 | 0.245282 | 0.002134 |
| ANKRD35 | 0.337696 | -0.12779 | -0.46549 | 0.003149 |
| ANKRD39 | -0.23449 | 0.04651 | 0.281 | 0.008413 |
| ANKRD40 | -0.50778 | -0.03239 | 0.47539 | 9.09E-05 |
| ANKRD50 | -0.25425 | 0.050205 | 0.304454 | 0.000228 |
| ANKS1A | 0.113196 | -0.08608 | -0.19928 | 0.005721 |
| ANKZF1 | 0.223038 | 0.061317 | -0.16172 | 0.009913 |
| ANLN | 0.22693 | -0.40584 | -0.63276 | 0.00084 |
| ANP32A | -0.4816 | -0.17984 | 0.301755 | 0.008116 |
| ANP32E | 0.670627 | -0.10058 | -0.7712 | 3.08E-05 |
| ANXA1 | 0.431624 | -0.02272 | -0.45434 | 0.000861 |
| ANXA3 | 0.247756 | -0.54275 | -0.79051 | 0.001424 |
| ANXA8 | 0.389824 | -0.56069 | -0.95051 | 0.000776 |
| ANXA9 | -0.63029 | 0.241705 | 0.871997 | 3.55E-06 |
| AP1G2 | -0.41966 | 0.183608 | 0.603272 | 4.03E-07 |
| AP1M2 | -0.42029 | 0.073904 | 0.494194 | 0.000489 |
| AP3B1 | -0.21774 | 0.031026 | 0.248764 | 2.76E-10 |
| AP3D1 | -0.17259 | 0.059962 | 0.232557 | 4.39E-05 |
| AP3M1 | -0.03461 | 0.229311 | 0.263918 | 0.006196 |
| AP3S1 | -0.14855 | 0.024438 | 0.172984 | 0.009962 |
| APBB2 | -0.27013 | 0.114381 | 0.384508 | 1.12E-06 |
| APEH | -0.13292 | 0.289303 | 0.422228 | 0.002147 |
| APEX2 | 0.322643 | -0.11115 | -0.43379 | 0.003688 |
| APOB | 0.382883 | -0.04181 | -0.42469 | 0.001377 |
| APP | 0.17543 | -0.31741 | -0.49284 | 0.002701 |
| APPL2 | -0.11964 | 0.276666 | 0.396308 | 0.000207 |
| AR | -1.03046 | 0.012457 | 1.042916 | 1.34E-09 |
| ARFGAP1 | -0.16074 | 0.038727 | 0.199465 | 0.009882 |
| ARFGAP2 | -0.14225 | 0.029686 | 0.171939 | 0.002244 |
| ARFGEF1 | -0.28679 | -0.01978 | 0.267013 | 0.002353 |
| ARFGEF3 | -0.78037 | 0.061459 | 0.841825 | 3.60E-07 |
| ARFIP1 | -0.14531 | 0.303572 | 0.448883 | 1.34E-06 |
| ARFIP2 | -0.47213 | 0.081561 | 0.553687 | 1.63E-05 |
| ARHGAP17 | 0.285954 | 0.116217 | -0.16974 | 0.002436 |
| ARHGAP21 | 0.27201 | -0.15787 | -0.42988 | 1.54E-05 |
| ARHGAP27 | 0.21927 | -0.02653 | -0.2458 | 0.001817 |
| ARHGAP31 | 0.248526 | 0.023607 | -0.22492 | 0.004774 |
| ARHGAP32 | -0.28285 | -0.02533 | 0.257526 | 0.004489 |
| ARHGAP35 | -0.32168 | 0.125857 | 0.447541 | 8.99E-07 |
| ARHGEF1 | 0.316807 | 0.055066 | -0.26174 | 1.46E-05 |
| ARHGEF10 | 0.346796 | 0.066154 | -0.28064 | 0.001557 |
| ARHGEF10L | 0.368329 | -0.11024 | -0.47857 | 5.88E-05 |
| ARHGEF16 | -0.39951 | 0.031219 | 0.430731 | 0.00154 |
| ARHGEF2 | 0.277296 | -0.14332 | -0.42061 | 1.16E-05 |
| ARID2 | -0.11856 | 0.079421 | 0.197977 | 0.000906 |
| ARL3 | -0.35497 | 0.271902 | 0.626876 | 2.32E-05 |
| ARL8A | 0.199979 | -0.06583 | -0.26581 | 0.007464 |
| ARMC9 | -0.05362 | 0.2893 | 0.342918 | 0.00291 |
| ARMT1 | -1.36505 | -0.47897 | 0.886084 | 8.28E-06 |
| ARPC3 | 0.293088 | -0.0095 | -0.30258 | 0.002731 |
| ARPC4 | 0.358336 | 0.115214 | -0.24312 | 0.001853 |
| ARRB1 | -0.28326 | 0.099357 | 0.382617 | 0.001084 |
| ARRDC1 | -0.17067 | 0.078231 | 0.248905 | 0.003675 |
| ASAH1 | -0.53669 | -0.03371 | 0.502971 | 0.000112 |
| ASL | -0.25796 | 0.110678 | 0.368638 | 1.50E-05 |
| ASMTL | -0.25599 | 0.113149 | 0.369142 | 0.000496 |
| ASNS | 0.417967 | -0.11198 | -0.52995 | 0.006314 |
| ASPN | -0.26988 | 0.289241 | 0.559124 | 0.002449 |
| ATAT1 | 0.015719 | -0.33148 | -0.3472 | 0.006239 |
| ATG16L1 | -0.18867 | 0.034296 | 0.222969 | 0.000799 |
| ATG2B | 0.000401 | 0.186097 | 0.185696 | 0.000395 |
| ATIC | -0.20965 | 0.046025 | 0.255674 | 0.006892 |
| ATL2 | 0.098632 | -0.41914 | -0.51777 | 0.000546 |
| ATOX1 | -0.29788 | 0.146118 | 0.444003 | 0.000631 |
| ATP11A | 0.25369 | -0.09587 | -0.34956 | 0.002145 |
| ATP1B3 | 0.258302 | -0.20464 | -0.46294 | 0.000841 |
| ATP8B1 | -0.67178 | -0.10953 | 0.562253 | 5.77E-05 |
| ATXN1 | -0.10725 | 0.158118 | 0.265369 | 0.00453 |
| AURKA | 0.242404 | -0.26802 | -0.51042 | 0.009249 |
| AVL9 | -0.18346 | 0.100598 | 0.284059 | 0.005374 |
| AZU1 | -0.38295 | -1.11218 | -0.72922 | 0.004611 |
| BAD | -0.45436 | -0.17542 | 0.27894 | 0.003941 |
| BAG1 | -0.28954 | 0.038968 | 0.328504 | 0.007005 |
| BAG2 | 0.430451 | -0.12918 | -0.55963 | 0.000894 |
| BAG3 | -0.42539 | -0.04534 | 0.380046 | 0.000333 |
| BANP | 0.271291 | 0.055276 | -0.21601 | 0.007315 |
| BAZ1B | 0.185203 | -0.18914 | -0.37435 | 0.000207 |
| BAZ2A | -0.17829 | 0.005667 | 0.183953 | 0.005327 |
| BBOX1 | 0.603411 | -0.3891 | -0.99251 | 0.000241 |
| BBS1 | -0.09941 | 0.266986 | 0.366399 | 0.000349 |
| BBS4 | -0.14721 | 0.170264 | 0.317474 | 0.002808 |
| BCAM | -0.42727 | 0.067465 | 0.49473 | 0.000143 |
| BCAS1 | -1.66484 | -0.79726 | 0.867577 | 5.01E-07 |
| BCAS3 | -0.13374 | 0.126074 | 0.25981 | 0.000231 |
| BCAT2 | -0.36843 | 0.011628 | 0.380063 | 0.000823 |
| BCL2 | -0.69147 | -0.08247 | 0.608997 | 0.003714 |
| BCL2L1 | -0.17252 | 0.126756 | 0.299276 | 0.007131 |
| BEND3 | 0.393883 | -0.08199 | -0.47587 | 0.003112 |
| BHLHE40 | -0.18437 | 0.134427 | 0.3188 | 0.008216 |
| BICD2 | 0.192595 | -0.03482 | -0.22741 | 0.000524 |
| BID | 0.274824 | 0.057818 | -0.21701 | 0.003687 |
| BIN3 | -0.2811 | 0.026917 | 0.308018 | 0.002441 |
| BIRC5 | 0.625411 | 0.032994 | -0.59242 | 0.005225 |
| BLOC1S4 | -0.00104 | 0.186124 | 0.187166 | 0.00739 |
| BLVRA | -0.58048 | 0.139741 | 0.720224 | 8.46E-07 |
| BOD1 | -0.22841 | 0.068044 | 0.296458 | 0.00113 |
| BOLA2 | -0.34031 | -0.07393 | 0.266383 | 0.006244 |
| BPI | -0.45542 | -1.31787 | -0.86245 | 0.005338 |
| BRD3 | -0.04197 | 0.0934 | 0.135367 | 0.009423 |
| BRK1 | 0.260334 | 0.011918 | -0.24842 | 3.31E-05 |
| BROX | -0.24392 | 0.105203 | 0.34912 | 0.000395 |
| BSG | 0.401677 | -0.13128 | -0.53295 | 0.000136 |
| BSPRY | -0.61934 | -0.12646 | 0.492886 | 0.000913 |
| BTF3 | -0.06466 | 0.182326 | 0.246984 | 0.002289 |
| BZW1 | -0.38895 | -0.17538 | 0.213565 | 0.009559 |
| C10orf76 | -0.09166 | 0.184326 | 0.275985 | 0.000807 |
| C11orf58 | -0.3115 | -0.01891 | 0.292595 | 0.00049 |
| C12orf10 | -0.19293 | 0.308574 | 0.501503 | 9.50E-06 |
| C12orf29 | -0.35361 | -0.05062 | 0.302989 | 0.001403 |
| C12orf57 | -0.1936 | 0.176571 | 0.370173 | 0.004709 |
| C16orf70 | 0.038663 | 0.287163 | 0.2485 | 0.000683 |
| C17orf75 | -0.23842 | 0.069539 | 0.307962 | 0.000327 |
| C1orf21 | -1.01199 | -0.45262 | 0.559369 | 0.004985 |
| C1orf226 | -0.4435 | -0.04033 | 0.403175 | 0.00291 |
| C1orf27 | -0.47671 | -0.06509 | 0.411626 | 1.50E-05 |
| C2orf88 | 0.313446 | -0.08645 | -0.39989 | 0.008829 |
| C3orf58 | 0.682569 | 0.074202 | -0.60837 | 2.33E-05 |
| C4BPA | 0.314545 | -0.12716 | -0.4417 | 0.005798 |
| C4BPB | 0.433591 | 0.052042 | -0.38155 | 0.005709 |
| C5orf30 | -0.25005 | 0.142453 | 0.392501 | 0.009435 |
| C9orf64 | -0.26472 | 0.081039 | 0.345754 | 1.03E-07 |
| CA12 | -1.40415 | -0.28257 | 1.121584 | 2.45E-08 |
| CA13 | 0.397544 | -0.20239 | -0.59994 | 0.001704 |
| CA8 | -0.90149 | -0.12412 | 0.777377 | 0.001155 |
| CAB39L | -0.28408 | 0.145234 | 0.429316 | 0.00167 |
| CADPS2 | -0.29448 | 0.168418 | 0.462899 | 0.000868 |
| CALB2 | 0.494724 | -0.25904 | -0.75376 | 0.009139 |
| CALCOCO2 | -0.38169 | -0.07781 | 0.303886 | 0.000344 |
| CALR | 0.05504 | -0.28082 | -0.33586 | 0.002856 |
| CALU | 0.33113 | -0.13074 | -0.46187 | 0.000942 |
| CAMK2G | 0.330135 | 0.019638 | -0.3105 | 0.00179 |
| CANT1 | -0.23839 | 0.136883 | 0.375275 | 0.001603 |
| CAP2 | -0.47001 | 0.056864 | 0.526871 | 0.003303 |
| CAPN13 | -1.17952 | -0.3332 | 0.846328 | 2.39E-06 |
| CAPN7 | -0.19111 | 0.042108 | 0.233219 | 2.39E-05 |
| CARD19 | -0.27814 | 0.084045 | 0.362188 | 0.000111 |
| CARD6 | 0.270767 | -0.07234 | -0.34311 | 0.005485 |
| CARD9 | 0.440062 | 0.135122 | -0.30494 | 0.004422 |
| CASP6 | -0.16503 | 0.122781 | 0.287808 | 0.000818 |
| CASP9 | -0.13841 | 0.132012 | 0.270417 | 0.006564 |
| CASZ1 | -0.54686 | -0.05932 | 0.487545 | 4.88E-05 |
| CBFB | 0.445468 | 0.100899 | -0.34457 | 6.79E-05 |
| CBL | 0.360472 | 0.159917 | -0.20055 | 0.004746 |
| CBR1 | 0.385867 | -0.05958 | -0.44544 | 0.000243 |
| CBR4 | -0.51092 | 0.169283 | 0.680207 | 1.15E-06 |
| CBS | 0.020244 | -0.98169 | -1.00193 | 0.000115 |
| CCDC112 | -0.50584 | 0.079214 | 0.585049 | 0.000149 |
| CCDC134 | 0.282929 | -0.17035 | -0.45328 | 0.000557 |
| CCDC88A | 0.169633 | -0.16373 | -0.33336 | 0.004891 |
| CCNB1 | 0.163589 | -0.23085 | -0.39444 | 0.009922 |
| CCNB2 | 0.411499 | -0.00759 | -0.41909 | 0.001316 |
| CCND1 | -0.50249 | 0.101409 | 0.603904 | 0.000283 |
| CD244 | -0.41001 | 0.143311 | 0.553319 | 0.000113 |
| CD38 | 0.073063 | -0.31321 | -0.38628 | 0.008938 |
| CD40 | 0.185788 | -0.31519 | -0.50098 | 0.007706 |
| CDC123 | 0.521319 | 0.179754 | -0.34157 | 0.002031 |
| CDC20 | 0.273614 | -0.4222 | -0.69581 | 0.000408 |
| CDC42BPB | -0.11527 | 0.084668 | 0.199935 | 0.000857 |
| CDC42EP1 | 0.41052 | 0.023252 | -0.38727 | 7.94E-05 |
| CDCA2 | 0.131558 | -0.51139 | -0.64295 | 0.002759 |
| CDCA5 | 0.565131 | 0.039978 | -0.52515 | 0.001344 |
| CDH3 | 0.629129 | -0.22658 | -0.85571 | 2.64E-06 |
| CDK17 | -0.31885 | -0.03969 | 0.279158 | 0.00171 |
| CDK19 | 0.306365 | -0.0835 | -0.38987 | 0.004447 |
| CDK6 | 0.546456 | -0.01966 | -0.56611 | 5.66E-05 |
| CDK9 | -0.18077 | 0.074779 | 0.255552 | 0.000222 |
| CDYL2 | -0.78128 | -0.15085 | 0.630426 | 3.06E-07 |
| CEACAM1 | 0.318287 | -0.35674 | -0.67503 | 0.006901 |
| CEBPB | 0.388136 | -0.16439 | -0.55252 | 1.01E-05 |
| CEBPG | 0.09287 | -0.44508 | -0.53795 | 0.008112 |
| CEBPZ | 0.183302 | -0.14104 | -0.32435 | 0.008521 |
| CELSR1 | -0.54093 | -0.00854 | 0.532394 | 6.54E-06 |
| CENPE | 0.351138 | -0.10294 | -0.45407 | 0.007504 |
| CENPF | 0.322558 | -0.1341 | -0.45665 | 0.000119 |
| CEP170 | 0.233748 | -0.06034 | -0.29409 | 0.000819 |
| CERS2 | -0.74764 | -0.35851 | 0.389132 | 0.003277 |
| CERS6 | -0.85568 | -0.14412 | 0.71156 | 9.81E-06 |
| CETN2 | -0.31199 | 0.038677 | 0.350667 | 0.00022 |
| CETN3 | -0.48904 | 0.058789 | 0.547825 | 7.61E-07 |
| CFAP73 | -0.50524 | -0.26326 | 0.241977 | 0.007358 |
| CHAD | -1.03161 | -0.47553 | 0.556076 | 0.003449 |
| CHCHD5 | -0.44356 | 0.176637 | 0.620199 | 8.66E-07 |
| CHCHD6 | -0.32394 | -0.02721 | 0.296736 | 0.000168 |
| CHD1L | 0.507471 | 0.117479 | -0.38999 | 0.000601 |
| CHD3 | -0.37112 | -0.05727 | 0.31385 | 5.16E-05 |
| CHEK1 | 0.25008 | -0.05278 | -0.30286 | 0.006167 |
| CHI3L1 | 0.686307 | -0.02993 | -0.71624 | 0.000516 |
| CHI3L2 | 0.656697 | 0.02766 | -0.62904 | 0.001301 |
| CIB1 | -0.4581 | -0.00485 | 0.453242 | 0.00173 |
| CILP | -0.54007 | -0.06527 | 0.4748 | 0.004872 |
| CIRBP | -0.50262 | -0.13157 | 0.371055 | 9.01E-05 |
| CISD1 | 0.263094 | -0.10755 | -0.37065 | 0.002506 |
| CIT | 0.137527 | -0.09154 | -0.22907 | 0.006265 |
| CKAP2 | 0.413574 | -0.10331 | -0.51689 | 0.00025 |
| CLASP2 | -0.1556 | -0.00021 | 0.155385 | 0.004859 |
| CLEC16A | -0.22801 | -0.05243 | 0.175586 | 0.003001 |
| CLGN | -1.20671 | -0.53974 | 0.666974 | 0.002715 |
| CLIC6 | -1.63558 | -0.94351 | 0.692064 | 0.006076 |
| CLINT1 | -0.39381 | -0.09021 | 0.303598 | 5.19E-07 |
| CLSPN | 0.17575 | -0.16564 | -0.34139 | 0.007206 |
| CLSTN2 | -0.96345 | -0.13575 | 0.827703 | 3.91E-06 |
| CLUAP1 | -0.19267 | 0.066997 | 0.259671 | 0.00295 |
| CLUH | 0.111983 | -0.23377 | -0.34575 | 0.002708 |
| CMBL | -1.48657 | -0.20387 | 1.282701 | 2.39E-13 |
| CNOT8 | -0.64125 | -0.14614 | 0.495114 | 0.00028 |
| COASY | -0.38078 | 0.045569 | 0.426351 | 0.000129 |
| COG1 | -0.21348 | 0.047251 | 0.260732 | 4.89E-05 |
| COG2 | -0.26344 | 0.055318 | 0.318757 | 0.000126 |
| COG3 | -0.24604 | 0.088106 | 0.334143 | 0.000277 |
| COG4 | -0.20275 | 0.036531 | 0.239279 | 0.002637 |
| COG5 | -0.22531 | -0.00156 | 0.223746 | 0.00143 |
| COG6 | -0.0528 | 0.160132 | 0.212928 | 0.009185 |
| COG7 | -0.12035 | 0.099357 | 0.219704 | 0.005132 |
| COG8 | -0.2191 | -0.00304 | 0.216057 | 0.002437 |
| COL11A1 | -0.68283 | -0.20488 | 0.477953 | 0.007084 |
| COL15A1 | 0.402938 | 0.086812 | -0.31613 | 0.006324 |
| COL4A2 | 0.41782 | -0.12322 | -0.54104 | 0.00016 |
| COL4A3BP | -0.22409 | 0.071923 | 0.296012 | 6.93E-06 |
| COLGALT1 | 0.350432 | -0.05963 | -0.41006 | 0.000113 |
| COPB2 | -0.1535 | 0.001898 | 0.155398 | 0.001621 |
| COPRS | -0.49872 | -0.08195 | 0.416768 | 0.000524 |
| COPS3 | -0.12495 | 0.026698 | 0.151651 | 0.000716 |
| COPS4 | 0.023685 | 0.199248 | 0.175562 | 0.001125 |
| COPS6 | 0.114471 | 0.30659 | 0.192119 | 0.008299 |
| COPS7A | -0.08031 | 0.150587 | 0.230895 | 0.00396 |
| COPS9 | -0.37193 | 0.186312 | 0.558244 | 0.000424 |
| COPZ1 | -0.11192 | 0.102724 | 0.214639 | 0.004468 |
| COQ10B | -0.34715 | -0.01928 | 0.327867 | 0.000864 |
| CORO2A | -0.55673 | -0.02485 | 0.53188 | 3.85E-07 |
| COTL1 | 0.252147 | -0.22263 | -0.47478 | 0.00231 |
| CPNE2 | 0.578637 | 0.220046 | -0.35859 | 0.000277 |
| CPT1A | -0.61422 | -0.08684 | 0.52738 | 5.69E-05 |
| CRABP1 | -0.07411 | -1.40574 | -1.33164 | 0.000494 |
| CRAT | -1.12586 | -0.1849 | 0.940965 | 1.73E-06 |
| CRBN | -0.25498 | 0.043567 | 0.298544 | 0.000422 |
| CREB3L4 | -0.40019 | 0.022057 | 0.422247 | 0.00781 |
| CREG1 | 0.537637 | 0.26118 | -0.27646 | 0.003969 |
| CRIP1 | -1.73579 | -0.63947 | 1.096317 | 6.85E-08 |
| CROCC | -0.34502 | -0.11686 | 0.228154 | 0.005008 |
| CROT | -0.92199 | -0.04859 | 0.873399 | 8.67E-10 |
| CRYAB | 0.508237 | -0.27174 | -0.77997 | 0.00085 |
| CSNK2A2 | 0.155773 | -0.0177 | -0.17347 | 0.007272 |
| CSPG4 | 0.276307 | -0.24499 | -0.52129 | 0.000229 |
| CTPS1 | 0.480686 | -0.12094 | -0.60163 | 2.41E-07 |
| CTPS2 | -0.12152 | 0.251819 | 0.373337 | 0.004829 |
| CTSC | 0.532118 | 0.081349 | -0.45077 | 0.000687 |
| CTSL | 0.478694 | 0.235043 | -0.24365 | 0.00909 |
| CTTNBP2NL | 0.216158 | -0.00366 | -0.21981 | 0.001895 |
| CTU2 | 0.376279 | 0.075293 | -0.30099 | 0.000659 |
| CUEDC1 | -0.29872 | 0.194175 | 0.492898 | 0.000351 |
| CUL1 | 0.137472 | -0.09276 | -0.23024 | 0.00267 |
| CUL2 | 0.242956 | 0.018156 | -0.2248 | 0.007609 |
| CUL3 | -0.09849 | 0.046332 | 0.144817 | 0.008057 |
| CXADR | 0.451862 | -0.10081 | -0.55268 | 0.001978 |
| CXXC5 | -0.7138 | -0.03709 | 0.676711 | 4.72E-09 |
| CXorf40A | -0.3314 | 0.180104 | 0.511501 | 0.003568 |
| CYB5A | -0.9455 | -0.11197 | 0.833528 | 1.58E-06 |
| CYB5R1 | -0.48584 | -0.02456 | 0.461282 | 0.000128 |
| CYB5R2 | 0.503007 | -0.09089 | -0.5939 | 0.000571 |
| CYB5R3 | 0.224356 | -0.02436 | -0.24871 | 0.005073 |
| CYCS | 0.358938 | 0.024144 | -0.33479 | 0.004791 |
| CYP27A1 | 0.178314 | -0.21909 | -0.39741 | 0.005417 |
| CYP4F11 | 0.092525 | -0.3276 | -0.42013 | 0.008772 |
| CYP4X1 | -0.63814 | -0.16998 | 0.468163 | 0.005676 |
| CYP4Z1 | -0.19037 | 0.181764 | 0.372131 | 0.001389 |
| CYP7B1 | 0.429933 | 0.049006 | -0.38093 | 0.001114 |
| DAB2IP | 0.246246 | -0.11912 | -0.36537 | 0.000624 |
| DACH1 | -0.97843 | -0.41642 | 0.562008 | 0.000485 |
| DAPK2 | 0.222031 | -0.17637 | -0.3984 | 0.004713 |
| DAPP1 | 0.419407 | 0.040314 | -0.37909 | 0.006976 |
| DAXX | 0.272129 | 0.009397 | -0.26273 | 0.001433 |
| DAZAP2 | -0.04971 | 0.384083 | 0.433798 | 0.000262 |
| DCAF11 | -0.25212 | 0.054684 | 0.306806 | 0.0013 |
| DCAF13 | 0.132852 | -0.11664 | -0.24949 | 0.009878 |
| DCAF5 | -0.44888 | -0.11463 | 0.334249 | 0.000342 |
| DCAF7 | -0.1328 | 0.07047 | 0.203269 | 0.002239 |
| DCAF8 | -0.30601 | -0.00572 | 0.300286 | 0.00038 |
| DCTN4 | 0.068757 | 0.235848 | 0.167091 | 0.003238 |
| DCTPP1 | -0.62123 | -0.09449 | 0.526748 | 0.001161 |
| DCXR | -0.84818 | -0.13687 | 0.71131 | 3.93E-07 |
| DDB1 | -0.01886 | 0.184899 | 0.203759 | 0.001237 |
| DDRGK1 | -0.4291 | -0.1697 | 0.259399 | 0.002124 |
| DDX11 | 0.222789 | -0.20028 | -0.42307 | 0.002545 |
| DDX19A | 0.359459 | -0.03219 | -0.39164 | 2.26E-05 |
| DDX21 | 0.065 | -0.26591 | -0.33091 | 0.009956 |
| DDX39A | 0.281422 | -0.06004 | -0.34146 | 0.004825 |
| DDX47 | 0.264278 | -0.03237 | -0.29665 | 0.003791 |
| DDX49 | 0.211962 | -0.10427 | -0.31623 | 0.001334 |
| DECR2 | -0.51429 | -0.05413 | 0.46016 | 0.000698 |
| DEFA3 | -1.13778 | -1.9709 | -0.83312 | 0.004128 |
| DEK | 0.324583 | -0.07361 | -0.39819 | 0.000143 |
| DENND2D | -0.44783 | -0.12726 | 0.32057 | 0.000938 |
| DENR | -0.12101 | 0.019654 | 0.14066 | 0.007257 |
| DGKE | -0.38887 | 0.106631 | 0.495503 | 0.00345 |
| DHCR24 | -0.82165 | -0.23432 | 0.587327 | 0.004117 |
| DHODH | 0.329099 | 0.07741 | -0.25169 | 0.005944 |
| DHRS3 | 0.490834 | -0.18117 | -0.672 | 1.96E-05 |
| DHRS4 | -0.3046 | 0.06337 | 0.367969 | 0.000322 |
| DHX29 | -0.20699 | -0.01046 | 0.196527 | 0.005557 |
| DHX40 | -0.07679 | 0.18388 | 0.26067 | 0.001773 |
| DIAPH2 | 0.424572 | 0.105803 | -0.31877 | 9.39E-05 |
| DIAPH3 | 0.374329 | -0.04868 | -0.42301 | 0.004981 |
| DIDO1 | -0.26575 | -0.00955 | 0.256194 | 1.55E-05 |
| DLAT | 0.386605 | 0.122679 | -0.26393 | 0.003392 |
| DLG3 | -0.366 | 0.081602 | 0.447601 | 2.14E-05 |
| DLGAP5 | 0.370656 | -0.1373 | -0.50796 | 0.000121 |
| DMD | 0.637589 | -0.02448 | -0.66207 | 3.76E-06 |
| DMKN | 0.520678 | -0.05043 | -0.57111 | 0.008609 |
| DMTN | 0.078858 | -0.36754 | -0.4464 | 0.005681 |
| DMXL1 | -0.11555 | 0.083894 | 0.199448 | 0.00178 |
| DNAJA4 | -0.4957 | -0.0978 | 0.397901 | 0.000632 |
| DNAJB4 | 0.366194 | 0.063669 | -0.30252 | 0.005127 |
| DNAJC1 | -1.17121 | -0.53373 | 0.637483 | 4.80E-06 |
| DNAJC12 | -1.68548 | -0.72908 | 0.956394 | 6.63E-08 |
| DNAJC3 | 0.172671 | -0.13015 | -0.30282 | 0.004944 |
| DNAL1 | -0.15729 | 0.205174 | 0.362462 | 0.000151 |
| DNALI1 | -0.63771 | -0.07873 | 0.558983 | 0.000225 |
| DNMBP | -0.08702 | 0.227851 | 0.314868 | 0.000744 |
| DNMT3A | 0.258305 | -0.15168 | -0.40998 | 0.000938 |
| DNPEP | -0.28061 | 0.028043 | 0.308656 | 0.001841 |
| DOCK7 | 0.213347 | -0.04719 | -0.26054 | 0.00214 |
| DOPEY1 | 0.301228 | -0.01596 | -0.31718 | 0.007614 |
| DOPEY2 | -0.39338 | -0.08778 | 0.305597 | 0.007648 |
| DPCD | -0.47058 | -0.07595 | 0.394628 | 0.00163 |
| DPP3 | -0.28437 | 0.031286 | 0.315653 | 0.001651 |
| DPP8 | -0.08715 | 0.161706 | 0.24886 | 0.003438 |
| DPP9 | -0.0395 | 0.200738 | 0.240236 | 0.001004 |
| DPYSL2 | 0.453199 | 0.165967 | -0.28723 | 0.004519 |
| DRG1 | 0.261817 | 0.046367 | -0.21545 | 0.006956 |
| DSC2 | 0.418547 | -0.43033 | -0.84888 | 2.90E-07 |
| DSC3 | 0.349766 | -0.07301 | -0.42277 | 0.004123 |
| DSG2 | 0.211793 | -0.09797 | -0.30976 | 0.005098 |
| DTWD2 | -0.23765 | 0.147343 | 0.384989 | 0.000693 |
| DTX3 | -0.04548 | 0.320843 | 0.366326 | 0.000313 |
| DTYMK | -0.13367 | 0.049927 | 0.183601 | 0.009825 |
| DUSP22 | 0.264778 | -0.2115 | -0.47628 | 0.001006 |
| DXO | 0.19672 | -0.07376 | -0.27048 | 0.004503 |
| DYNLL2 | -0.43985 | -0.01244 | 0.427409 | 0.001335 |
| DYNLT1 | 0.344578 | 0.051715 | -0.29286 | 0.000363 |
| DYSF | 0.385603 | 0.033432 | -0.35217 | 0.00213 |
| EBF1 | 0.153973 | -0.14287 | -0.29685 | 0.009496 |
| ECHDC2 | -0.27979 | 0.092946 | 0.372735 | 0.002263 |
| ECI1 | -0.06592 | 0.246986 | 0.312902 | 0.007672 |
| ECI2 | -0.72118 | 0.048502 | 0.769687 | 6.77E-06 |
| ECM1 | -0.23676 | 0.180692 | 0.417447 | 0.002979 |
| ECT2 | 0.374621 | -0.09896 | -0.47358 | 0.000646 |
| EDARADD | -0.79102 | 0.174939 | 0.965958 | 2.34E-07 |
| EDC4 | 0.249268 | 0.047663 | -0.2016 | 0.000445 |
| EDEM2 | 0.473978 | 0.197116 | -0.27686 | 0.009335 |
| EEF1A2 | -1.14396 | -0.05679 | 1.087178 | 5.53E-06 |
| EEF1D | 0.058513 | -0.18035 | -0.23886 | 0.006542 |
| EEF2K | -0.16711 | 0.057086 | 0.224194 | 0.007913 |
| EFHD1 | -0.33537 | 0.124091 | 0.459464 | 0.009767 |
| EFNA5 | 0.09671 | -0.41901 | -0.51572 | 0.001853 |
| EGFR | 0.133876 | -0.76762 | -0.9015 | 2.33E-05 |
| EHBP1 | 0.308961 | -0.03495 | -0.34391 | 1.44E-06 |
| EHD1 | 0.356115 | 0.057759 | -0.29836 | 0.000254 |
| EIF4G3 | -0.26427 | -0.0809 | 0.183366 | 0.006035 |
| ELANE | -0.51801 | -1.14143 | -0.62342 | 0.004177 |
| ELAVL2 | 0.512284 | -0.05123 | -0.56352 | 0.00021 |
| ELK1 | 0.258955 | -0.05728 | -0.31624 | 0.003437 |
| ELMSAN1 | -0.1225 | 0.019298 | 0.141797 | 0.004313 |
| ELOVL5 | -0.88676 | -0.2669 | 0.619866 | 0.000553 |
| ELP2 | -0.14212 | 0.086454 | 0.22857 | 0.000573 |
| EMC2 | 0.216609 | 0.019872 | -0.19674 | 0.001542 |
| EML2 | -0.45677 | -0.15824 | 0.29853 | 0.006707 |
| EML4 | 0.081876 | -0.25909 | -0.34096 | 0.001672 |
| EMSY | -0.26687 | -0.04898 | 0.217884 | 0.002137 |
| EN1 | 0.356887 | -0.05488 | -0.41177 | 0.005692 |
| ENO1 | 0.446306 | 0.036469 | -0.40984 | 0.001152 |
| ENPP1 | -0.74917 | -0.14633 | 0.602841 | 5.48E-05 |
| ENSA | -0.26597 | 0.015285 | 0.281259 | 0.001459 |
| ENTPD5 | -0.67358 | -0.16328 | 0.510304 | 0.003123 |
| EOGT | 0.297759 | -0.04439 | -0.34215 | 5.58E-05 |
| EPB41 | 0.125053 | -0.15476 | -0.27981 | 0.003865 |
| EPB41L5 | -0.61462 | -0.0685 | 0.54612 | 6.36E-06 |
| EPHB3 | 0.501282 | -0.38696 | -0.88824 | 1.01E-05 |
| EPM2AIP1 | -0.16073 | 0.117271 | 0.278003 | 0.004306 |
| EPN2 | -0.14938 | 0.20092 | 0.350302 | 0.001426 |
| EPN3 | -0.54846 | -0.02928 | 0.519181 | 0.000364 |
| EPS15L1 | -0.02663 | 0.267516 | 0.29415 | 0.000212 |
| EPS8L1 | -0.50005 | -0.09084 | 0.409211 | 0.009707 |
| ERBB2 | -1.16331 | -0.56294 | 0.60037 | 0.000298 |
| ERBB3 | -0.49436 | -0.06772 | 0.426646 | 9.67E-05 |
| ERBB4 | -1.21316 | -0.37705 | 0.836106 | 0.003084 |
| ERBIN | -0.2328 | -0.00846 | 0.224345 | 0.001375 |
| ERGIC1 | -1.10672 | -0.33956 | 0.767162 | 6.50E-07 |
| ERI1 | 0.348198 | 0.078945 | -0.26925 | 0.007521 |
| ERICH5 | 0.352047 | -0.13249 | -0.48454 | 0.002109 |
| ERP44 | 0.321099 | 0.019249 | -0.30185 | 2.23E-05 |
| ERRFI1 | 0.321797 | -0.11791 | -0.43971 | 0.009373 |
| ESR1 | -1.38119 | -0.18211 | 1.199076 | 1.22E-07 |
| ESS2 | 0.239451 | 0.058879 | -0.18057 | 0.007991 |
| ETFDH | -0.25504 | 0.040294 | 0.295333 | 0.006088 |
| ETNK2 | -0.32606 | 0.045745 | 0.371807 | 0.006224 |
| EVL | -0.73617 | -0.07456 | 0.661615 | 1.51E-05 |
| EXD2 | -0.35505 | -0.09955 | 0.255498 | 0.006223 |
| EXOC1 | -0.06778 | 0.09146 | 0.159238 | 0.00223 |
| EXOC2 | -0.03363 | 0.107843 | 0.141469 | 0.002377 |
| EXOC5 | -0.10883 | 0.078783 | 0.187617 | 0.000158 |
| EXOC6 | -0.23536 | 0.14351 | 0.378868 | 2.72E-09 |
| EXOC7 | 0.04025 | 0.252065 | 0.211815 | 0.000737 |
| EXOC8 | -0.1463 | 0.051348 | 0.197652 | 4.33E-05 |
| EXT2 | 0.499483 | 0.173173 | -0.32631 | 0.002694 |
| EXTL2 | 0.488291 | 0.146617 | -0.34167 | 0.001396 |
| EYA3 | 0.125828 | -0.14613 | -0.27196 | 0.001431 |
| F9 | 0.457157 | -0.00574 | -0.4629 | 0.000346 |
| FAAH | -0.71563 | 0.126344 | 0.841972 | 6.57E-06 |
| FABP5 | 0.472445 | -0.16607 | -0.63851 | 0.000268 |
| FABP7 | -0.67041 | -2.04748 | -1.37707 | 0.00087 |
| FAF1 | 0.281684 | 0.024194 | -0.25749 | 0.008249 |
| FAF2 | -0.38461 | 0.044526 | 0.429138 | 2.21E-05 |
| FAH | -0.52828 | -0.01319 | 0.515082 | 0.000737 |
| FAHD1 | -0.09689 | 0.33346 | 0.430352 | 4.41E-05 |
| FAM102A | -0.32866 | 0.108615 | 0.437276 | 2.66E-07 |
| FAM114A2 | -0.40681 | -0.03282 | 0.373998 | 1.78E-06 |
| FAM120A | -0.29225 | 0.00785 | 0.300098 | 9.72E-08 |
| FAM126B | 0.23355 | -0.0104 | -0.24395 | 0.003996 |
| FAM172A | -0.20591 | 0.211393 | 0.417305 | 2.71E-05 |
| FAM173A | -0.25343 | 0.153633 | 0.407068 | 0.002221 |
| FAM198B | -0.48104 | 0.069623 | 0.550665 | 6.37E-05 |
| FAM208A | -0.07509 | 0.072543 | 0.147632 | 0.006098 |
| FAM213B | -0.26409 | 0.031917 | 0.296007 | 0.00088 |
| FAM241A | -0.63278 | -0.18685 | 0.445937 | 0.009839 |
| FAM49A | 0.500355 | 0.075072 | -0.42528 | 0.001741 |
| FAM83B | -0.12672 | -0.65088 | -0.52416 | 0.009525 |
| FAM83H | -0.40152 | -0.07679 | 0.324729 | 0.001893 |
| FAM84B | -0.58641 | -0.0121 | 0.57431 | 7.95E-05 |
| FAM98A | 0.283263 | 0.044806 | -0.23846 | 0.00042 |
| FAM98B | -0.04304 | 0.154296 | 0.197338 | 0.004735 |
| FAM98C | -0.36374 | 0.155318 | 0.519054 | 3.73E-06 |
| FAP | -0.10379 | 0.22706 | 0.330849 | 0.007209 |
| FASN | -1.02564 | -0.32364 | 0.701997 | 9.60E-05 |
| FAT1 | 0.292296 | -0.10222 | -0.39451 | 4.92E-07 |
| FBLIM1 | 0.315882 | -0.13364 | -0.44952 | 0.000334 |
| FBP1 | -0.89955 | 0.051619 | 0.951171 | 1.27E-06 |
| FBP2 | -1.0763 | -0.43978 | 0.636514 | 0.001709 |
| FBXL6 | -0.34513 | 0.034954 | 0.380081 | 0.002499 |
| FBXO22 | -0.0694 | 0.228317 | 0.297714 | 0.006921 |
| FBXO38 | -0.35445 | 0.098322 | 0.452776 | 6.85E-09 |
| FCN3 | 0.349242 | 0.010611 | -0.33863 | 0.008278 |
| FDXR | -0.49287 | -0.10832 | 0.384549 | 0.000375 |
| FERMT1 | 0.605795 | -0.03139 | -0.63719 | 9.02E-05 |
| FES | 0.363519 | 0.061803 | -0.30172 | 0.002123 |
| FGB | 0.288074 | -0.08582 | -0.37389 | 0.008299 |
| FGD3 | -0.57144 | -0.19813 | 0.373314 | 0.007236 |
| FGD6 | -0.20937 | 0.040022 | 0.249396 | 0.003168 |
| FGF2 | 0.099211 | -0.64934 | -0.74855 | 0.000344 |
| FKBP3 | -0.18958 | 0.006657 | 0.196238 | 0.009231 |
| FKBP4 | -0.71199 | -0.14544 | 0.566554 | 0.000153 |
| FLII | -0.12141 | 0.097655 | 0.219065 | 2.70E-05 |
| FLNB | -0.57653 | -0.20311 | 0.373423 | 0.002857 |
| FLT1 | 0.423056 | 0.011186 | -0.41187 | 0.001894 |
| FLYWCH2 | -0.5668 | -0.00355 | 0.563252 | 5.14E-08 |
| FMN1 | -0.35954 | 0.026602 | 0.386142 | 0.001776 |
| FMNL2 | 0.102084 | -0.24999 | -0.35207 | 0.00951 |
| FNDC3B | 0.215601 | -0.16799 | -0.3836 | 0.000223 |
| FOLH1 | 0.459178 | -0.0014 | -0.46058 | 0.000246 |
| FOLR1 | 0.314677 | -0.1421 | -0.45678 | 0.001435 |
| FOXA1 | -1.14134 | 0.176323 | 1.317666 | 1.31E-09 |
| FOXC1 | 0.41969 | -0.18649 | -0.60618 | 0.000502 |
| FOXP1 | -0.28481 | 0.171325 | 0.456133 | 1.93E-05 |
| FPGT | -0.15701 | 0.11777 | 0.274781 | 0.00067 |
| FRMD4A | 0.254917 | -0.128 | -0.38292 | 0.002159 |
| FRY | -0.28728 | 0.057623 | 0.344899 | 0.00203 |
| FSCN1 | 0.336408 | -0.14207 | -0.47848 | 1.57E-05 |
| FSTL3 | -0.55567 | -0.01549 | 0.540173 | 0.003143 |
| FUBP3 | -0.10746 | 0.064793 | 0.172255 | 0.002006 |
| FUT8 | -0.59692 | 0.054542 | 0.651465 | 1.33E-05 |
| FYCO1 | -0.41715 | 0.091275 | 0.508426 | 4.18E-07 |
| GAB1 | -0.0967 | 0.15033 | 0.247034 | 0.009623 |
| GABARAPL2 | 0.592955 | 0.095397 | -0.49756 | 0.000633 |
| GAK | -0.16651 | 0.043305 | 0.209811 | 0.000558 |
| GALE | -0.27982 | 0.121256 | 0.401079 | 0.006516 |
| GALNT10 | -0.32026 | 0.047647 | 0.367911 | 0.009094 |
| GALNT5 | -0.34123 | 0.222103 | 0.563333 | 0.000146 |
| GALNT6 | -0.66357 | 0.176194 | 0.839768 | 2.81E-08 |
| GALNT7 | -0.70307 | 0.003118 | 0.706189 | 4.03E-08 |
| GAMT | -0.74036 | 0.194495 | 0.934854 | 9.03E-06 |
| GARS | 0.305214 | 0.062849 | -0.24236 | 0.006222 |
| GATA3 | -1.12946 | 0.039942 | 1.169406 | 5.23E-13 |
| GBF1 | -0.07267 | 0.155593 | 0.228268 | 0.002082 |
| GBP1 | 0.097942 | -0.4101 | -0.50804 | 0.009694 |
| GCC2 | -0.31266 | -0.01265 | 0.300009 | 6.33E-07 |
| GDAP1 | -0.61743 | -0.25434 | 0.363088 | 0.008336 |
| GDI2 | 0.42909 | 0.106757 | -0.32233 | 0.008781 |
| GDPD1 | -0.43956 | -0.13685 | 0.302702 | 0.004984 |
| GEMIN8 | -0.35446 | -0.01685 | 0.337608 | 0.005538 |
| GFER | -0.31456 | 0.085705 | 0.400262 | 0.000682 |
| GFPT2 | 0.345194 | -0.01046 | -0.35565 | 0.005525 |
| GFRA1 | -1.824 | -0.52026 | 1.303744 | 1.72E-09 |
| GGCT | -0.46031 | 0.033597 | 0.493907 | 7.85E-05 |
| GGPS1 | -0.20872 | 0.203803 | 0.412524 | 0.000232 |
| GHDC | -0.57518 | -0.13099 | 0.444194 | 6.54E-06 |
| GIGYF2 | -0.0787 | 0.04503 | 0.123725 | 0.002915 |
| GIMAP6 | 0.371791 | -0.10235 | -0.47414 | 0.002899 |
| GLB1L | 0.380088 | -0.09187 | -0.47196 | 0.001474 |
| GLCCI1 | -0.05675 | 0.239021 | 0.295773 | 0.005201 |
| GLI3 | -0.66275 | -0.15162 | 0.511131 | 0.000773 |
| GLIPR1 | 0.324808 | 0.030471 | -0.29434 | 0.004282 |
| GLIPR2 | 0.557012 | -0.03745 | -0.59446 | 5.26E-05 |
| GLOD5 | -0.24997 | 0.132646 | 0.382617 | 0.002073 |
| GLS | 0.753895 | -0.09576 | -0.84966 | 3.75E-07 |
| GLUD1 | -0.19889 | 0.191306 | 0.390194 | 0.001785 |
| GMPPA | -0.17778 | 0.075 | 0.252778 | 0.002175 |
| GMPR2 | -0.34768 | 0.133727 | 0.481403 | 9.59E-06 |
| GNA13 | -0.16164 | 0.080224 | 0.241868 | 0.001613 |
| GNPNAT1 | -0.35536 | 0.146189 | 0.501552 | 0.000376 |
| GOLGA1 | -0.38702 | -0.05579 | 0.331232 | 5.51E-05 |
| GOLGA2 | -0.31037 | -0.03829 | 0.272089 | 6.85E-05 |
| GOLGB1 | -0.3948 | -0.13671 | 0.258085 | 0.002398 |
| GOLM1 | -0.67476 | -0.11761 | 0.557143 | 7.26E-05 |
| GOLPH3L | -0.37706 | 0.080138 | 0.457203 | 1.75E-05 |
| GORASP1 | -0.16722 | 0.178714 | 0.34593 | 0.000235 |
| GP2 | -1.64948 | -1.00739 | 0.642085 | 0.001296 |
| GPATCH1 | 0.288964 | 0.125796 | -0.16317 | 0.005527 |
| GPD1L | -0.63909 | -0.00871 | 0.630384 | 0.000217 |
| GPD2 | -0.37752 | -0.08563 | 0.291898 | 0.009954 |
| GPSM2 | 0.333408 | -0.02714 | -0.36054 | 0.006326 |
| GPT2 | 0.713389 | -0.15513 | -0.86852 | 1.47E-05 |
| GREB1 | -1.10105 | -0.23352 | 0.86753 | 1.97E-05 |
| GRHL1 | 0.53291 | -0.05779 | -0.5907 | 0.000256 |
| GRIPAP1 | -0.09344 | 0.090295 | 0.183733 | 0.001803 |
| GSDMD | -0.1645 | 0.143956 | 0.308452 | 0.005228 |
| GSKIP | -0.03521 | 0.288078 | 0.323284 | 0.00991 |
| GSPT1 | -0.3552 | -0.09714 | 0.258052 | 0.003745 |
| GSS | -0.14453 | 0.242686 | 0.387219 | 1.18E-05 |
| GSTCD | -0.36726 | 0.068315 | 0.435574 | 6.49E-05 |
| GSTM3 | -1.47306 | -0.63621 | 0.836853 | 1.93E-05 |
| GSTP1 | 0.339819 | -0.16859 | -0.50841 | 0.001596 |
| GSTZ1 | -0.39925 | 0.04665 | 0.445898 | 0.000652 |
| GTF2I | 0.36579 | -0.07111 | -0.4369 | 0.00014 |
| GUF1 | -0.07934 | 0.192726 | 0.272062 | 0.006532 |
| HACD3 | -0.74676 | -0.07442 | 0.672342 | 5.99E-06 |
| HAGH | -0.3033 | -0.02427 | 0.279031 | 0.000668 |
| HAPLN3 | 0.620214 | -0.21132 | -0.83154 | 0.000258 |
| HARS | -0.11354 | 0.14276 | 0.256298 | 0.001088 |
| HARS2 | -0.13051 | 0.201498 | 0.332012 | 6.56E-05 |
| HAUS2 | 0.232312 | -0.06949 | -0.3018 | 4.52E-05 |
| HAUS6 | 0.302312 | -0.03827 | -0.34058 | 7.92E-05 |
| HAUS7 | 0.226176 | -0.02321 | -0.24938 | 0.008017 |
| HAUS8 | 0.268374 | -0.05743 | -0.32581 | 0.002408 |
| HDAC11 | -0.61618 | -0.16454 | 0.451638 | 0.000261 |
| HDGFL3 | -0.95313 | -0.27446 | 0.678667 | 0.000319 |
| HEATR6 | -0.82703 | -0.31594 | 0.511095 | 0.000495 |
| HEBP1 | -1.02476 | -0.55405 | 0.470708 | 0.002999 |
| HECTD4 | -0.322 | 0.038838 | 0.36084 | 5.42E-07 |
| HEXIM1 | -0.3688 | 0.124281 | 0.493084 | 1.07E-05 |
| HGD | -1.17389 | -0.27258 | 0.901307 | 3.68E-05 |
| HID1 | -0.94671 | -0.02231 | 0.924391 | 1.36E-07 |
| HIF1AN | -0.14155 | 0.230779 | 0.372324 | 3.59E-07 |
| HIST1H1A | 0.327959 | -0.22356 | -0.55152 | 0.001834 |
| HK1 | -0.26333 | 0.018474 | 0.281802 | 0.001315 |
| HK3 | 0.277823 | -0.18507 | -0.46289 | 0.002038 |
| HMCES | 0.294201 | 0.014731 | -0.27947 | 0.005776 |
| HMCN1 | -0.13729 | 0.234103 | 0.371389 | 0.000679 |
| HMGCL | -0.25989 | 0.037103 | 0.296989 | 0.000482 |
| HMGCS2 | -1.65355 | -0.58172 | 1.071831 | 7.97E-05 |
| HMGN5 | -0.76677 | -0.16422 | 0.602547 | 8.38E-05 |
| HNMT | -0.29452 | 0.048157 | 0.342672 | 0.000705 |
| HOMER1 | 0.39576 | 0.040301 | -0.35546 | 0.006056 |
| HOOK3 | 0.28974 | -0.00553 | -0.29527 | 0.000558 |
| HPGDS | -0.39907 | 0.130342 | 0.529411 | 0.001282 |
| HPSE | 0.242034 | -0.06648 | -0.30852 | 0.006067 |
| HSD11B1 | 0.31728 | 0.024368 | -0.29291 | 0.005953 |
| HSD17B4 | -0.31463 | 0.008417 | 0.323043 | 0.001352 |
| HSD17B8 | -0.50484 | 0.041197 | 0.546033 | 1.10E-05 |
| HSD3B7 | -0.51734 | -0.04986 | 0.467483 | 8.48E-05 |
| HSDL2 | -0.34012 | 0.088146 | 0.428264 | 0.001334 |
| HSP90B1 | 0.15775 | -0.09826 | -0.25601 | 0.00172 |
| HSPA14 | 0.364195 | 0.053674 | -0.31052 | 0.001423 |
| HSPA2 | -0.61459 | -0.12966 | 0.48493 | 0.008479 |
| HSPA4 | -0.42943 | -0.08378 | 0.345648 | 3.70E-06 |
| HSPB1 | -0.75602 | -0.0884 | 0.667623 | 0.00017 |
| HSPBAP1 | 0.289951 | -0.00587 | -0.29582 | 0.002542 |
| HTRA1 | -0.35714 | 0.016177 | 0.373318 | 0.0064 |
| HYOU1 | 0.100256 | -0.21386 | -0.31411 | 0.006512 |
| IARS2 | -0.09823 | 0.125505 | 0.223735 | 0.006852 |
| ICA1 | -0.31344 | -0.02 | 0.293434 | 0.006585 |
| ICAM1 | 0.418842 | -0.16292 | -0.58176 | 0.00073 |
| ID4 | 0.12111 | -0.08777 | -0.20888 | 0.002406 |
| IDE | -0.08473 | 0.115246 | 0.199979 | 0.003718 |
| IFI16 | 0.34472 | -0.00387 | -0.34859 | 0.001209 |
| IFRD1 | 0.590707 | -0.20224 | -0.79295 | 6.01E-06 |
| IFT122 | -0.15946 | 0.223434 | 0.382897 | 0.00021 |
| IFT140 | -0.38782 | 0.110366 | 0.498184 | 9.37E-08 |
| IFT172 | -0.05032 | 0.212437 | 0.262757 | 0.000127 |
| IFT20 | -0.17923 | 0.274935 | 0.454167 | 0.008528 |
| IFT22 | -0.09987 | 0.267328 | 0.367201 | 0.000517 |
| IFT27 | -0.19701 | 0.220387 | 0.417398 | 0.001148 |
| IFT43 | -0.39107 | 0.053284 | 0.444353 | 2.90E-06 |
| IFT46 | -0.41237 | 0.06389 | 0.476263 | 0.000386 |
| IFT52 | -0.11843 | 0.222334 | 0.340764 | 0.001228 |
| IFT74 | -0.22987 | 0.170988 | 0.400858 | 0.000408 |
| IFT80 | -0.10306 | 0.134163 | 0.237225 | 0.009345 |
| IFT81 | -0.42548 | -0.04459 | 0.380891 | 4.58E-06 |
| IGF2BP2 | 0.414317 | -0.43194 | -0.84626 | 0.00074 |
| IGFBP2 | -1.00336 | -0.36167 | 0.641689 | 4.47E-05 |
| IGFBP4 | -0.5888 | -0.14141 | 0.447387 | 0.0013 |
| IKBKB | 0.006645 | 0.229868 | 0.223224 | 0.000668 |
| IKBKE | 0.435718 | 0.119134 | -0.31658 | 0.004644 |
| IL6ST | -0.57374 | 0.032796 | 0.60654 | 1.26E-05 |
| ILVBL | -0.12609 | 0.137128 | 0.263222 | 0.001304 |
| IMP3 | 0.473279 | 0.101603 | -0.37168 | 1.50E-05 |
| IMPA2 | 0.172068 | -0.48051 | -0.65258 | 0.001726 |
| IMPDH2 | -0.11446 | 0.194793 | 0.309251 | 0.005901 |
| INCENP | 0.432327 | -0.13861 | -0.57094 | 1.93E-05 |
| ING2 | -0.35963 | 0.073208 | 0.432841 | 4.06E-05 |
| ING3 | 0.246902 | -0.05922 | -0.30612 | 0.000486 |
| INPP1 | 0.338753 | 0.045594 | -0.29316 | 0.002602 |
| INPP4B | -1.03847 | 0.09049 | 1.12896 | 9.34E-09 |
| INPP5J | -0.28856 | 0.176955 | 0.46552 | 0.002206 |
| INTS13 | 0.301039 | 0.109088 | -0.19195 | 0.001719 |
| INTS6L | 0.319595 | -0.10521 | -0.4248 | 0.005166 |
| IQGAP3 | 0.377814 | -0.00233 | -0.38014 | 0.001602 |
| IQSEC1 | -0.18037 | 0.090503 | 0.270874 | 0.000236 |
| IRS1 | -0.57125 | -0.0309 | 0.540353 | 2.65E-06 |
| IRX5 | -0.69954 | -0.1667 | 0.53284 | 0.000639 |
| ISLR | -0.18918 | 0.237148 | 0.426324 | 0.004877 |
| ISOC1 | -0.34678 | 0.179785 | 0.526564 | 0.000397 |
| ITGA6 | 0.567791 | -0.32013 | -0.88792 | 2.18E-05 |
| ITGB5 | -0.31239 | 0.010521 | 0.322908 | 0.006674 |
| ITIH4 | 0.451869 | 0.10256 | -0.34931 | 0.003366 |
| ITPK1 | -0.40198 | 0.209619 | 0.6116 | 5.10E-05 |
| ITPKB | 0.181036 | -0.12051 | -0.30154 | 0.009088 |
| IVD | -0.50192 | -0.03402 | 0.4679 | 4.80E-05 |
| JMJD7 | -0.09603 | 0.24733 | 0.343361 | 0.009707 |
| JPT2 | -0.733 | -0.18405 | 0.548947 | 2.70E-06 |
| JUN | 0.420356 | 0.100453 | -0.3199 | 0.005824 |
| KANK1 | 0.696585 | 0.000827 | -0.69576 | 3.39E-09 |
| KARS | 0.276265 | 0.02037 | -0.25589 | 0.001676 |
| KATNA1 | 0.304979 | 0.065032 | -0.23995 | 0.009025 |
| KBTBD4 | -0.19041 | 0.053338 | 0.243745 | 0.008316 |
| KCTD14 | 0.380956 | -0.44059 | -0.82155 | 0.000125 |
| KCTD20 | 0.205969 | -0.18429 | -0.39026 | 0.003557 |
| KCTD21 | -0.07905 | 0.307326 | 0.386373 | 0.003425 |
| KCTD3 | -0.57754 | -0.16949 | 0.408048 | 1.05E-05 |
| KCTD6 | -0.65903 | 0.046622 | 0.705649 | 9.60E-05 |
| KCTD9 | 0.332703 | -0.02573 | -0.35844 | 0.001996 |
| KDM3A | 0.242319 | -0.13088 | -0.3732 | 2.60E-05 |
| KDM3B | -0.01568 | 0.135618 | 0.151297 | 0.004175 |
| KDM4B | -0.27225 | 0.038898 | 0.31115 | 0.001415 |
| KERA | -0.39975 | 0.133764 | 0.533517 | 0.002805 |
| KHDRBS3 | 0.333996 | -0.35062 | -0.68462 | 1.45E-05 |
| KIAA1191 | -0.30962 | 0.005105 | 0.314726 | 0.004821 |
| KIAA1211 | -0.42806 | 0.105602 | 0.533663 | 0.001229 |
| KIAA1211L | -0.60273 | -0.28458 | 0.318154 | 0.007688 |
| KIAA2013 | 0.24553 | -0.1128 | -0.35833 | 0.001796 |
| KIF11 | 0.194421 | -0.1487 | -0.34312 | 0.006636 |
| KIF13A | -0.18461 | 0.080704 | 0.265314 | 0.000327 |
| KIF13B | -0.49556 | -0.08281 | 0.412748 | 0.000571 |
| KIF14 | 0.536859 | -0.13766 | -0.67452 | 3.77E-05 |
| KIF15 | 0.179493 | -0.17501 | -0.3545 | 0.000513 |
| KIF16B | -0.69248 | -0.15592 | 0.536553 | 1.13E-07 |
| KIF18B | 0.518636 | -0.01671 | -0.53535 | 0.007395 |
| KIF1A | 0.308341 | -0.16757 | -0.47591 | 0.006579 |
| KIF1BP | -0.12147 | 0.188085 | 0.309559 | 0.001018 |
| KIF22 | 0.348548 | 0.062598 | -0.28595 | 0.00629 |
| KIF23 | 0.133338 | -0.27558 | -0.40892 | 0.005491 |
| KIF2C | 0.360124 | -0.19028 | -0.55041 | 0.000198 |
| KIF3A | -0.24529 | 0.06832 | 0.313612 | 3.69E-06 |
| KIF3B | -0.20707 | -0.02763 | 0.179436 | 0.004064 |
| KIF4A | 0.194581 | -0.14144 | -0.33602 | 0.003015 |
| KIFAP3 | -0.1069 | 0.152089 | 0.258994 | 0.000287 |
| KIFC1 | 0.174791 | -0.303 | -0.47779 | 0.000521 |
| KIN | 0.21211 | -0.11933 | -0.33144 | 0.001466 |
| KLHDC2 | -0.26117 | 0.074228 | 0.3354 | 0.002177 |
| KLHDC7A | -0.59929 | -0.06596 | 0.53333 | 0.002399 |
| KLHL36 | 0.356038 | 0.084806 | -0.27123 | 0.002058 |
| KLHL7 | 0.340383 | 0.056671 | -0.28371 | 0.004568 |
| KLK10 | 0.297334 | -0.07899 | -0.37632 | 0.009067 |
| KRT16 | -0.43829 | -1.79456 | -1.35627 | 4.88E-05 |
| KRT17 | 0.127375 | -1.43434 | -1.56171 | 1.07E-05 |
| KRT18 | -1.17111 | -0.07186 | 1.099247 | 1.86E-06 |
| KRT19 | -1.08297 | -0.37196 | 0.711014 | 2.23E-05 |
| KRT23 | 0.33356 | -0.94629 | -1.27985 | 1.32E-05 |
| KRT5 | -0.19932 | -1.27815 | -1.07883 | 0.000913 |
| KRT6B | -0.21678 | -1.21705 | -1.00026 | 0.000463 |
| KRT8 | -1.04219 | -0.09422 | 0.947971 | 6.03E-07 |
| KRT81 | -0.28934 | -1.66396 | -1.37462 | 0.000406 |
| KTI12 | 0.28631 | 0.071771 | -0.21454 | 0.003298 |
| KTN1 | -0.51766 | -0.10728 | 0.410381 | 1.04E-06 |
| LAD1 | -0.28037 | -0.88528 | -0.60491 | 0.007423 |
| LAMA5 | 0.372205 | -0.20766 | -0.57987 | 5.21E-05 |
| LAMB1 | 0.423684 | -0.00854 | -0.43222 | 0.000792 |
| LAMC1 | 0.410886 | -0.03889 | -0.44977 | 0.00112 |
| LAMC2 | -0.08531 | -0.88512 | -0.79981 | 0.000275 |
| LAMP3 | 0.403228 | -0.28296 | -0.68619 | 0.000394 |
| LARP1 | -0.24118 | 0.000408 | 0.241585 | 6.32E-05 |
| LASP1 | -0.59748 | -0.33721 | 0.260267 | 0.009148 |
| LBR | 0.322367 | -0.24296 | -0.56533 | 0.000263 |
| LCN2 | -0.25116 | -1.16002 | -0.90886 | 0.003152 |
| LCP1 | 0.467917 | 0.036293 | -0.43162 | 0.009929 |
| LDHB | 0.77564 | -0.22764 | -1.00328 | 8.40E-14 |
| LEF1 | -0.6514 | -0.12632 | 0.525076 | 0.001252 |
| LGALS8 | -0.15789 | 0.08835 | 0.246237 | 0.002032 |
| LGALSL | 0.434597 | -0.02238 | -0.45698 | 8.78E-05 |
| LIMA1 | -0.5689 | -0.04263 | 0.526271 | 1.94E-06 |
| LIMK2 | 0.332244 | -0.00415 | -0.3364 | 1.07E-05 |
| LIN7A | -0.53751 | 0.007864 | 0.54537 | 0.002489 |
| LIX1L | 0.270154 | -0.04308 | -0.31324 | 0.009137 |
| LLGL2 | -0.36189 | 0.102624 | 0.464516 | 0.00011 |
| LMO4 | 0.341525 | -0.24705 | -0.58857 | 0.000467 |
| LMX1B | -0.58982 | 0.109132 | 0.698949 | 3.24E-05 |
| LOXL4 | 0.292801 | -0.07242 | -0.36522 | 0.006105 |
| LPIN1 | 0.510132 | -0.1192 | -0.62934 | 7.50E-06 |
| LRBA | -0.62749 | 0.033053 | 0.660542 | 2.80E-07 |
| LRRC17 | -0.51559 | 0.049575 | 0.565168 | 0.001481 |
| LRRC20 | 0.026496 | 0.29578 | 0.269284 | 0.003362 |
| LRRC42 | -0.07361 | -0.39451 | -0.32091 | 0.007897 |
| LRRFIP1 | -0.49437 | -0.11858 | 0.375791 | 8.90E-08 |
| LRSAM1 | -0.13772 | 0.243119 | 0.380843 | 1.63E-05 |
| LTBP1 | 0.238474 | -0.22276 | -0.46123 | 0.001733 |
| LY75 | 0.292328 | -0.24025 | -0.53258 | 0.000127 |
| LYAR | 0.123181 | -0.27405 | -0.39723 | 0.006933 |
| LYN | 0.54352 | -0.09001 | -0.63354 | 8.13E-06 |
| LYPLAL1 | -0.10036 | 0.178116 | 0.278478 | 0.002773 |
| LYZ | -0.01482 | -0.5298 | -0.51498 | 0.001955 |
| LZTFL1 | -0.50958 | 0.020239 | 0.529815 | 6.43E-07 |
| MADD | -0.32588 | -0.08587 | 0.240004 | 0.002673 |
| MAGED2 | -0.96878 | -0.26874 | 0.700043 | 3.98E-08 |
| MAGED4 | 0.407316 | -0.09568 | -0.503 | 0.002098 |
| MAN2A1 | -0.16969 | 0.148019 | 0.317707 | 0.000186 |
| MAN2B1 | 0.293374 | 0.104647 | -0.18873 | 0.00669 |
| MAN2B2 | -0.03711 | 0.291946 | 0.32906 | 0.002475 |
| MAP2 | 0.128364 | -0.71192 | -0.84029 | 0.000296 |
| MAP2K3 | 0.193975 | -0.04238 | -0.23635 | 0.003304 |
| MAP2K4 | -0.18541 | 0.083248 | 0.268657 | 0.001853 |
| MAP3K6 | 0.277871 | 0.012554 | -0.26532 | 0.004189 |
| MAP4K4 | 0.245404 | -0.07525 | -0.32065 | 0.003858 |
| MAP7D3 | 0.329221 | -0.02569 | -0.35491 | 0.003281 |
| MAP9 | -0.62915 | -0.22183 | 0.40732 | 0.009271 |
| MAPK3 | -0.02561 | 0.163687 | 0.189297 | 0.009349 |
| MAPK9 | -0.20988 | 0.260308 | 0.470186 | 1.72E-05 |
| MAPKAPK2 | -0.21387 | -0.04356 | 0.170306 | 0.009798 |
| MAPRE2 | 0.437673 | -0.04003 | -0.47771 | 0.001295 |
| MAPT | -1.71694 | -0.47985 | 1.237097 | 8.56E-09 |
| MASP1 | 0.379123 | 0.080692 | -0.29843 | 0.007539 |
| MASTL | 0.24099 | -0.22001 | -0.461 | 0.005926 |
| MAT2A | -0.07626 | 0.091559 | 0.167821 | 0.000714 |
| MATK | 0.12763 | -0.15504 | -0.28267 | 0.002426 |
| MATN2 | 0.071451 | -0.38599 | -0.45744 | 0.00917 |
| MATR3 | -0.15128 | 0.013356 | 0.164636 | 0.001066 |
| MAVS | -0.2407 | -0.00573 | 0.234971 | 0.007906 |
| MB | -0.86188 | -0.31496 | 0.546913 | 0.00462 |
| MBNL2 | 0.200818 | -0.21717 | -0.41799 | 0.003056 |
| MBOAT7 | -0.83154 | -0.26151 | 0.570027 | 0.000267 |
| MCAM | 0.577085 | -0.06142 | -0.63851 | 1.62E-06 |
| MCC | -0.19105 | 0.074804 | 0.265853 | 0.006574 |
| MCCC2 | -0.75574 | -0.11443 | 0.641309 | 6.65E-07 |
| MCM2 | 0.213507 | -0.13565 | -0.34915 | 0.008856 |
| MCM3 | 0.14831 | -0.24042 | -0.38873 | 0.004302 |
| MCM4 | 0.307386 | -0.0643 | -0.37169 | 0.006344 |
| MCM5 | 0.226616 | -0.16697 | -0.39359 | 0.008138 |
| MCM6 | 0.218511 | -0.17821 | -0.39672 | 0.004921 |
| MCM7 | 0.222382 | -0.13257 | -0.35495 | 0.009884 |
| MDH1 | 0.052859 | -0.14062 | -0.19347 | 0.005469 |
| MDN1 | 0.275058 | -0.0758 | -0.35086 | 0.001053 |
| MDP1 | -0.17547 | 0.223056 | 0.398526 | 7.49E-05 |
| ME2 | 0.592954 | 0.190017 | -0.40294 | 0.000335 |
| MELTF | 0.638471 | -0.21694 | -0.85541 | 0.000909 |
| METTL14 | -0.18311 | 0.056305 | 0.239418 | 0.000948 |
| METTL26 | -0.48287 | 0.054459 | 0.537333 | 1.45E-09 |
| METTL3 | -0.12007 | 0.068721 | 0.188794 | 0.004089 |
| MEX3A | 0.349318 | -0.13073 | -0.48005 | 1.79E-05 |
| MFGE8 | 0.586347 | -0.06182 | -0.64817 | 0.000918 |
| MGAT4A | -0.28478 | 0.075879 | 0.360662 | 0.000907 |
| MGST2 | -0.46715 | -0.15442 | 0.31273 | 0.002261 |
| MIA | 0.623692 | -0.53364 | -1.15733 | 1.76E-05 |
| MIA3 | -0.50943 | -0.19485 | 0.314584 | 0.000571 |
| MICAL3 | 0.274593 | -0.04328 | -0.31787 | 0.000249 |
| MICALL1 | 0.32465 | -0.26628 | -0.59093 | 8.55E-06 |
| MID1 | 0.241346 | -0.2817 | -0.52305 | 0.000431 |
| MID1IP1 | -0.41589 | -0.00809 | 0.407802 | 0.001107 |
| MIEN1 | -1.03303 | -0.2037 | 0.829335 | 8.02E-07 |
| MIF | -0.63365 | -0.17209 | 0.461566 | 0.008132 |
| MINDY1 | -0.50293 | -0.0004 | 0.502531 | 1.25E-05 |
| MINPP1 | 0.048963 | 0.330831 | 0.281868 | 0.00199 |
| MIPEP | -0.33226 | -0.04727 | 0.28499 | 0.00241 |
| MIS12 | -0.33863 | -0.13209 | 0.206536 | 0.009018 |
| MISP | -0.99675 | -0.2987 | 0.698045 | 4.12E-05 |
| MKI67 | 0.213016 | -0.32422 | -0.53724 | 0.001811 |
| MKL2 | -0.54784 | -0.08325 | 0.464586 | 1.15E-06 |
| MLKL | 0.314826 | -0.00165 | -0.31647 | 0.002114 |
| MLPH | -1.21458 | 0.034925 | 1.249503 | 6.77E-13 |
| MLX | 0.370641 | 0.063539 | -0.3071 | 0.003459 |
| MMAB | -0.30666 | 0.011853 | 0.318511 | 0.009021 |
| MMACHC | 0.395509 | 0.001983 | -0.39353 | 1.06E-05 |
| MMP14 | 0.35162 | 0.094631 | -0.25699 | 0.009126 |
| MMP9 | -0.14551 | -0.8235 | -0.67799 | 0.003627 |
| MOB1A | 0.086234 | -0.12613 | -0.21236 | 0.009602 |
| MOB3B | 0.190504 | -0.05669 | -0.24719 | 0.004112 |
| MOCS2 | -0.30943 | 0.089044 | 0.398474 | 0.000161 |
| MOCS3 | -0.19613 | 0.268 | 0.464134 | 1.88E-06 |
| MOGS | 0.332077 | 0.02509 | -0.30699 | 0.00041 |
| MON1B | 0.404752 | 0.124647 | -0.2801 | 0.000329 |
| MORC2 | 0.218794 | -0.06156 | -0.28035 | 0.002587 |
| MPI | -0.19683 | 0.253949 | 0.450777 | 0.000266 |
| MPND | -0.09352 | 0.180654 | 0.274179 | 0.006169 |
| MPO | 0.08304 | -0.62613 | -0.70917 | 0.000681 |
| MPP6 | 0.538926 | -0.09097 | -0.62989 | 7.41E-08 |
| MPP7 | -0.37645 | 0.0405 | 0.416949 | 0.002672 |
| MRAS | 0.534525 | -0.06411 | -0.59863 | 0.000149 |
| MREG | -0.37907 | 0.127343 | 0.506411 | 2.30E-05 |
| MRFAP1 | -0.01982 | 0.328441 | 0.348257 | 0.009098 |
| MRPL40 | -0.05981 | 0.12395 | 0.183759 | 0.002761 |
| MRPL54 | -0.07384 | 0.170748 | 0.244586 | 0.006641 |
| MSH6 | 0.303041 | -0.18106 | -0.4841 | 0.001199 |
| MSI2 | -0.63024 | 0.064848 | 0.695092 | 4.31E-07 |
| MSLN | 0.840678 | -0.08363 | -0.92431 | 1.37E-06 |
| MSN | 0.250287 | -0.15746 | -0.40774 | 0.005674 |
| MSRA | 0.172265 | -0.1419 | -0.31416 | 0.000553 |
| MSX2 | -0.23015 | 0.235726 | 0.465877 | 0.006584 |
| MTA3 | -0.10165 | 0.206839 | 0.308489 | 0.008085 |
| MTHFD1L | 0.662964 | -0.32554 | -0.98851 | 2.87E-08 |
| MTMR2 | 0.391549 | 0.057288 | -0.33426 | 0.000247 |
| MTX3 | -0.29502 | -0.0169 | 0.278116 | 0.000349 |
| MUC5B | -1.32718 | -0.73189 | 0.595295 | 0.006978 |
| MXRA8 | -0.42986 | 0.059393 | 0.489252 | 0.003477 |
| MYB | -0.15645 | 0.280141 | 0.436588 | 0.005846 |
| MYO1D | -0.09742 | 0.22212 | 0.319545 | 0.007727 |
| MYO1E | 0.398262 | 0.063977 | -0.33429 | 0.000177 |
| MYO5C | -0.52183 | 0.014516 | 0.536347 | 1.13E-06 |
| MYO6 | -0.50241 | -0.08419 | 0.418216 | 0.000158 |
| MYO9B | 0.181628 | 0.013361 | -0.16827 | 0.008692 |
| MYOF | -0.40859 | -0.02959 | 0.379006 | 2.34E-05 |
| N4BP3 | -0.48046 | 0.11966 | 0.600122 | 5.92E-07 |
| N6AMT1 | -0.31769 | 0.134978 | 0.452666 | 1.09E-05 |
| NAA35 | -0.03836 | 0.14537 | 0.18373 | 0.002879 |
| NAA38 | -0.08937 | 0.204497 | 0.293871 | 0.00816 |
| NAB1 | 0.361325 | -0.043 | -0.40433 | 0.000857 |
| NABP1 | 0.379523 | -0.03959 | -0.41912 | 0.002796 |
| NABP2 | -0.13248 | 0.134386 | 0.266865 | 0.001709 |
| NADK2 | -0.24697 | 0.037869 | 0.284839 | 0.00299 |
| NAMPT | 0.324511 | -0.16719 | -0.4917 | 0.001826 |
| NANS | -0.39299 | 0.014901 | 0.407894 | 0.000539 |
| NAP1L4 | -0.15095 | 0.100244 | 0.251198 | 0.00883 |
| NAPG | -0.10384 | 0.111714 | 0.215555 | 0.004741 |
| NARFL | -0.10301 | 0.146086 | 0.249094 | 0.000229 |
| NAT1 | -1.43831 | -0.12743 | 1.31088 | 1.62E-06 |
| NAV1 | -0.34994 | -0.09688 | 0.253065 | 0.008522 |
| NAV2 | 0.361863 | -0.00574 | -0.3676 | 0.001831 |
| NBAS | -0.04598 | 0.093101 | 0.139084 | 0.004938 |
| NCALD | 0.545209 | -0.17624 | -0.72145 | 0.003113 |
| NCAM2 | -1.31571 | -0.77427 | 0.541441 | 0.000396 |
| NCAPD2 | 0.388042 | -0.04315 | -0.43119 | 0.002209 |
| NCAPG2 | 0.226785 | -0.09059 | -0.31738 | 0.001379 |
| NCAPH | 0.248439 | -0.1295 | -0.37794 | 0.00319 |
| NCK1 | 0.333901 | 0.074243 | -0.25966 | 0.002155 |
| NCK2 | 0.449178 | -0.11678 | -0.56596 | 3.26E-07 |
| NCKAP5L | 0.226198 | -0.02979 | -0.25599 | 0.004508 |
| NCS1 | 0.710109 | 0.149653 | -0.56046 | 0.000801 |
| NDC80 | 0.310016 | -0.10564 | -0.41566 | 0.000892 |
| NDRG1 | 0.358032 | -0.30387 | -0.6619 | 0.000148 |
| NDRG2 | 0.459617 | -0.46961 | -0.92922 | 4.76E-05 |
| NDUFA10 | -0.08076 | 0.170121 | 0.25088 | 0.000718 |
| NDUFA8 | -0.22175 | 0.002138 | 0.223887 | 0.008988 |
| NDUFAF2 | -0.31707 | -0.05492 | 0.262151 | 0.000932 |
| NDUFAF7 | 0.407233 | 0.084844 | -0.32239 | 5.85E-05 |
| NDUFC2 | -0.50909 | -0.20151 | 0.307576 | 0.004178 |
| NDUFS4 | -0.23277 | 0.002678 | 0.235449 | 0.004933 |
| NECAP1 | -0.26155 | 0.028274 | 0.289824 | 0.003721 |
| NEDD4 | -0.16623 | 0.111717 | 0.277944 | 0.004521 |
| NEDD4L | -0.56525 | -0.09009 | 0.475158 | 0.000105 |
| NEK9 | -0.20139 | 0.086179 | 0.287568 | 0.000235 |
| NES | 0.164623 | -0.42552 | -0.59014 | 0.003259 |
| NFIB | 0.875338 | -0.30139 | -1.17672 | 1.42E-08 |
| NFIC | -0.3005 | -0.0316 | 0.268905 | 0.001165 |
| NFIX | 0.496246 | -0.18143 | -0.67767 | 4.06E-06 |
| NFKBIE | 0.186406 | -0.08739 | -0.2738 | 0.005318 |
| NHSL1 | 0.331613 | -0.20507 | -0.53669 | 1.37E-06 |
| NID1 | 0.524786 | 0.13881 | -0.38598 | 0.002202 |
| NIFK | 0.164033 | -0.09172 | -0.25575 | 0.007287 |
| NIT1 | -0.16128 | 0.072072 | 0.233352 | 0.002152 |
| NKIRAS2 | -0.20492 | -0.0038 | 0.201112 | 0.003937 |
| NKRF | 0.166426 | -0.20975 | -0.37617 | 0.005663 |
| NLRC4 | 0.292735 | -0.0481 | -0.34084 | 0.007176 |
| NME1 | -0.67041 | -0.25023 | 0.420188 | 0.00736 |
| NME3 | -0.41309 | -0.03079 | 0.382303 | 0.000229 |
| NMT2 | 0.452096 | 0.030214 | -0.42188 | 9.90E-05 |
| NOL11 | 0.191363 | -0.0694 | -0.26076 | 0.004452 |
| NOL9 | 0.239467 | 0.063972 | -0.17549 | 0.006843 |
| NOS1AP | -0.72204 | -0.28146 | 0.44058 | 0.008041 |
| NOSTRIN | -0.89789 | -0.29292 | 0.604972 | 0.000209 |
| NOXA1 | -0.33433 | 0.11395 | 0.44828 | 1.35E-05 |
| NPEPPS | -0.54279 | -0.11692 | 0.425873 | 0.000134 |
| NPRL2 | -0.13504 | 0.167991 | 0.303031 | 0.000406 |
| NRAS | 0.523686 | 0.147454 | -0.37623 | 0.004448 |
| NSF | -0.34018 | -0.07399 | 0.266191 | 0.000243 |
| NSFL1C | -0.26841 | -0.05958 | 0.208832 | 0.006061 |
| NT5C | -0.25448 | 0.004985 | 0.259464 | 0.005169 |
| NT5C2 | 0.381355 | -0.10422 | -0.48557 | 2.83E-06 |
| NT5DC2 | 0.274213 | -0.17759 | -0.4518 | 0.004536 |
| NUBP2 | -0.0567 | 0.293217 | 0.349912 | 0.001151 |
| NUBPL | -0.37036 | -0.01214 | 0.358213 | 1.43E-05 |
| NUCB2 | -0.59785 | -0.09216 | 0.50569 | 0.000275 |
| NUDCD1 | 0.226688 | -0.0555 | -0.28219 | 0.008683 |
| NUDCD2 | -0.15352 | 0.118463 | 0.271981 | 0.002962 |
| NUDT12 | -0.94486 | -0.22509 | 0.719769 | 8.15E-09 |
| NUDT16L1 | -0.31066 | 0.178307 | 0.488965 | 2.80E-05 |
| NUDT3 | 0.208935 | -0.11254 | -0.32148 | 4.96E-05 |
| NUDT4 | -0.22388 | 0.303936 | 0.527821 | 1.77E-05 |
| NUDT9 | -0.11276 | 0.093141 | 0.205905 | 0.002816 |
| NUF2 | 0.24874 | -0.14414 | -0.39288 | 0.009885 |
| NUFIP2 | -0.28159 | -0.04772 | 0.233872 | 0.00095 |
| NUMA1 | -0.49487 | -0.07641 | 0.418466 | 4.01E-07 |
| OMD | -0.69701 | 0.085202 | 0.782216 | 0.000387 |
| OPHN1 | 0.380712 | -0.0698 | -0.45051 | 0.000191 |
| OSBPL1A | 0.267397 | -0.05088 | -0.31827 | 0.001041 |
| OSBPL6 | -0.18777 | 0.315679 | 0.503445 | 0.000412 |
| OSCP1 | -0.54682 | 0.123394 | 0.670217 | 5.93E-05 |
| OTUB1 | -0.19379 | 0.01976 | 0.213545 | 0.005064 |
| OTUD7B | -0.07503 | 0.129561 | 0.204593 | 0.002537 |
| OXCT1 | 0.307981 | -0.10201 | -0.40999 | 0.00018 |
| P4HTM | -0.32759 | 0.059484 | 0.387076 | 0.001235 |
| PAAF1 | -0.24853 | 0.188852 | 0.437383 | 1.68E-07 |
| PACSIN3 | 0.27724 | -0.06736 | -0.3446 | 0.006347 |
| PADI2 | 0.606586 | -0.5057 | -1.11228 | 1.10E-05 |
| PADI4 | -0.53007 | -1.18149 | -0.65142 | 0.0099 |
| PAFAH1B3 | -0.49977 | -0.02584 | 0.473926 | 7.86E-06 |
| PAK1IP1 | 0.271593 | -0.00556 | -0.27715 | 0.002137 |
| PANX1 | 0.231912 | -0.12194 | -0.35386 | 0.0056 |
| PAOX | -0.09369 | 0.176749 | 0.270441 | 0.007337 |
| PAPOLG | 0.252002 | -0.05532 | -0.30732 | 0.004469 |
| PAPSS1 | 0.268258 | -0.18115 | -0.44941 | 0.002152 |
| PARD6B | -0.41767 | 0.187928 | 0.605601 | 0.000132 |
| PATL1 | 0.289676 | 0.046032 | -0.24364 | 0.003544 |
| PAXX | -0.20805 | 0.141385 | 0.349434 | 1.25E-06 |
| PBLD | -0.26237 | 0.185389 | 0.44776 | 0.002205 |
| PCBD2 | -0.33442 | 0.167198 | 0.501614 | 7.26E-06 |
| PCBP3 | 0.555782 | 0.099053 | -0.45673 | 0.001659 |
| PCBP4 | 0.472481 | -0.08799 | -0.56047 | 0.000669 |
| PCDH1 | -0.26947 | 0.10824 | 0.377708 | 0.001375 |
| PCID2 | 0.154967 | -0.05842 | -0.21339 | 0.004153 |
| PCK2 | -0.29347 | 0.097607 | 0.39108 | 0.000562 |
| PCM1 | -0.61553 | -0.32116 | 0.29437 | 0.004234 |
| PCYOX1L | -0.24404 | 0.037855 | 0.281896 | 0.003028 |
| PDCD6IP | -0.20394 | -0.0008 | 0.203144 | 0.002185 |
| PDE12 | -0.0361 | 0.173868 | 0.209971 | 0.002452 |
| PDE4DIP | -0.09008 | 0.102135 | 0.192211 | 0.001203 |
| PDGFRB | -0.11588 | 0.164434 | 0.280311 | 0.008233 |
| PDIA6 | 0.298831 | -0.11098 | -0.40981 | 0.005543 |
| PDK1 | 0.61799 | 0.060002 | -0.55799 | 3.59E-05 |
| PDPR | 0.354746 | 0.095341 | -0.2594 | 0.004717 |
| PDXDC1 | -0.39495 | 0.17637 | 0.571322 | 0.00015 |
| PDZK1 | -1.61302 | -0.88793 | 0.725093 | 0.001977 |
| PEG3 | 0.160642 | -0.69802 | -0.85867 | 0.000518 |
| PEX19 | -0.13933 | 0.068014 | 0.207349 | 0.007893 |
| PEX7 | -0.17859 | 0.170824 | 0.349412 | 0.000751 |
| PFAS | -0.13861 | 0.081194 | 0.219803 | 0.000393 |
| PGD | 0.122327 | -0.14979 | -0.27211 | 0.009824 |
| PGM1 | 0.26918 | 0.012086 | -0.25709 | 0.003379 |
| PGP | -0.24773 | 0.057292 | 0.30502 | 0.001682 |
| PGPEP1 | -0.24985 | 0.316451 | 0.566301 | 6.59E-05 |
| PGR | -1.3258 | -0.44897 | 0.876832 | 5.73E-05 |
| PHGDH | 0.690203 | -0.34312 | -1.03332 | 1.09E-09 |
| PHPT1 | -0.40698 | 0.10825 | 0.515235 | 2.45E-05 |
| PHYHD1 | -0.5458 | -0.10062 | 0.44518 | 0.005353 |
| PIK3AP1 | 0.363906 | -0.12936 | -0.49327 | 8.54E-05 |
| PIK3CD | 0.284357 | 0.006246 | -0.27811 | 0.00862 |
| PIK3R2 | -0.14525 | 0.029257 | 0.174504 | 0.002005 |
| PIP4K2B | -0.20749 | 0.155348 | 0.362838 | 0.00383 |
| PIP4K2C | -0.30026 | 0.05532 | 0.355584 | 0.002701 |
| PITPNB | 0.284116 | 0.074176 | -0.20994 | 0.002042 |
| PKIB | -1.70873 | -0.47671 | 1.23202 | 9.77E-06 |
| PKNOX1 | -0.01042 | 0.228227 | 0.238645 | 0.008096 |
| PKP1 | 0.004488 | -0.91311 | -0.9176 | 0.000952 |
| PLA2G16 | -0.27762 | 0.377406 | 0.655028 | 1.80E-05 |
| PLA2G4A | 0.143508 | -0.82669 | -0.9702 | 1.81E-05 |
| PLA2G7 | 0.383437 | -0.12995 | -0.51338 | 0.000945 |
| PLAA | 0.136396 | -0.11374 | -0.25014 | 0.001772 |
| PLAT | -1.06072 | -0.54349 | 0.517231 | 0.006808 |
| PLAUR | 0.387261 | -0.10382 | -0.49108 | 1.34E-05 |
| PLCB4 | 0.402546 | -0.04661 | -0.44916 | 0.004852 |
| PLCG2 | 0.668712 | 0.102937 | -0.56577 | 8.98E-07 |
| PLCH1 | 0.463515 | 0.021305 | -0.44221 | 0.000144 |
| PLD3 | 0.480567 | 0.119017 | -0.36155 | 0.008108 |
| PLEKHF2 | -0.4238 | 0.078991 | 0.502791 | 1.09E-09 |
| PLEKHG1 | 0.417086 | -0.21678 | -0.63387 | 7.67E-06 |
| PLEKHG4B | 0.382291 | 0.005681 | -0.37661 | 0.007408 |
| PLIN2 | 0.237416 | -0.45247 | -0.68989 | 0.000377 |
| PLIN3 | -0.18176 | 0.028055 | 0.209819 | 0.004245 |
| PLOD1 | 0.548204 | -0.0074 | -0.55561 | 8.62E-05 |
| PLOD3 | 0.371063 | -0.03253 | -0.4036 | 0.000954 |
| PLS1 | -0.68045 | -0.10544 | 0.575004 | 0.001151 |
| PM20D2 | 0.421121 | -0.63283 | -1.05395 | 3.50E-09 |
| PMS1 | 0.221876 | -0.04047 | -0.26235 | 0.003049 |
| PNPLA4 | -0.22424 | 0.193231 | 0.417472 | 0.004656 |
| PNPO | -0.20729 | 0.22547 | 0.43276 | 0.000219 |
| POGLUT1 | 0.205007 | -0.1004 | -0.30541 | 0.001602 |
| POLB | -0.21855 | 0.099368 | 0.317919 | 0.003192 |
| POLR1B | 0.175825 | -0.12018 | -0.296 | 0.00277 |
| POLR1E | 0.238943 | -0.1822 | -0.42114 | 0.001971 |
| POSTN | -0.42752 | 0.166663 | 0.594182 | 0.000377 |
| PPDPF | -0.45196 | 0.028675 | 0.480639 | 0.007645 |
| PPM1A | -0.06378 | 0.111613 | 0.175388 | 0.002894 |
| PPM1F | 0.362916 | 0.084559 | -0.27836 | 0.000591 |
| PPM1H | -0.44842 | -0.0133 | 0.435115 | 0.003211 |
| PPME1 | -0.23077 | 0.057656 | 0.288428 | 0.004826 |
| PPP1CB | 0.221342 | 0.05946 | -0.16188 | 0.00805 |
| PPP1R14C | 0.462718 | -0.58275 | -1.04547 | 8.36E-08 |
| PPP1R37 | -0.22664 | 0.038845 | 0.265488 | 0.000265 |
| PPP1R7 | 0.003153 | 0.237003 | 0.233849 | 0.005253 |
| PPP1R9B | -0.32667 | -0.07943 | 0.247238 | 0.00077 |
| PPP2R3A | 0.345799 | -0.04641 | -0.39221 | 5.40E-05 |
| PPP2R5D | 0.30342 | -0.00145 | -0.30487 | 0.00116 |
| PPT1 | -0.12897 | 0.152144 | 0.281114 | 0.003696 |
| PREPL | 0.00024 | 0.191253 | 0.191013 | 0.002202 |
| PREX1 | -0.62544 | 0.082883 | 0.708324 | 6.07E-08 |
| PRKAA1 | 0.238524 | 0.052324 | -0.1862 | 0.001938 |
| PRKAR1B | 0.433889 | -0.01338 | -0.44727 | 0.001274 |
| PRKCD | -0.21122 | 0.020946 | 0.232162 | 0.000848 |
| PRKCZ | -0.47 | -0.10158 | 0.368417 | 0.000395 |
| PRKD3 | 0.463639 | 0.021773 | -0.44187 | 4.96E-05 |
| PRMT5 | -0.13258 | 0.028489 | 0.161064 | 0.009548 |
| PRODH | -1.15083 | -0.5324 | 0.618431 | 0.002319 |
| PROM1 | -0.41952 | -1.32654 | -0.90702 | 0.000557 |
| PROS1 | 0.330484 | -0.07716 | -0.40764 | 0.00582 |
| PROSER1 | 0.403577 | 0.080159 | -0.32342 | 0.000403 |
| PRPF18 | 0.292624 | 0.005274 | -0.28735 | 0.0025 |
| PRR15 | -1.32478 | -0.25825 | 1.066529 | 5.49E-07 |
| PRRC1 | -0.41312 | 0.038425 | 0.451544 | 2.39E-08 |
| PRSS23 | -1.2191 | -0.78572 | 0.433375 | 0.006773 |
| PRTFDC1 | 0.565525 | -0.38435 | -0.94988 | 7.18E-08 |
| PRTN3 | -0.47548 | -1.15937 | -0.68389 | 0.004379 |
| PSAT1 | 0.8169 | -0.57682 | -1.39372 | 2.82E-08 |
| PSIP1 | 0.244457 | -0.2666 | -0.51106 | 0.002215 |
| PSME4 | 0.266088 | -0.14637 | -0.41245 | 0.00073 |
| PTBP2 | 0.010846 | -0.69651 | -0.70736 | 0.004296 |
| PTEN | -0.0174 | 0.289709 | 0.307111 | 0.005374 |
| PTGES3 | -0.41131 | -0.09271 | 0.318599 | 0.000855 |
| PTGR2 | -0.35773 | 0.148707 | 0.506438 | 2.63E-05 |
| PTGS2 | -0.47349 | -1.4269 | -0.95341 | 0.000845 |
| PTK7 | 0.222645 | -0.14239 | -0.36504 | 0.008193 |
| PTP4A1 | 0.210499 | -0.05022 | -0.26071 | 0.006115 |
| PTP4A2 | -0.41973 | -0.04822 | 0.371506 | 0.001909 |
| PTPA | -0.27406 | 0.034013 | 0.308072 | 5.42E-05 |
| PTPN14 | 0.218805 | -0.10091 | -0.31971 | 0.000406 |
| PTPN2 | 0.228046 | -0.09545 | -0.32349 | 0.000103 |
| PTPN23 | -0.1209 | 0.081642 | 0.202546 | 0.000113 |
| PTRH1 | -0.11922 | 0.112758 | 0.231977 | 0.007342 |
| PTRH2 | -0.65834 | -0.25989 | 0.398441 | 0.000811 |
| PTX3 | 0.483682 | -0.72916 | -1.21284 | 3.57E-06 |
| PUM3 | 0.409845 | -0.10597 | -0.51581 | 0.000286 |
| PURA | -0.28647 | -0.00519 | 0.28128 | 0.001126 |
| PUS7 | 0.358063 | -0.14605 | -0.50411 | 0.000435 |
| PVR | 0.369149 | -0.1332 | -0.50235 | 5.77E-05 |
| PWWP2A | 0.449077 | 0.095157 | -0.35392 | 0.005669 |
| PYCARD | -0.50329 | 0.123105 | 0.626393 | 0.005588 |
| PYCR3 | -0.2541 | 0.046264 | 0.30036 | 0.001664 |
| PYM1 | -0.66152 | -0.12765 | 0.533873 | 1.20E-08 |
| QDPR | -0.39977 | 0.038127 | 0.437893 | 0.001839 |
| QKI | 0.373866 | 0.122075 | -0.25179 | 0.000311 |
| R3HDM2 | -0.30127 | 0.016304 | 0.317571 | 0.001686 |
| RAB11FIP3 | -0.1598 | 0.229373 | 0.389177 | 8.05E-05 |
| RAB11FIP4 | -0.48366 | 0.025129 | 0.508792 | 0.000413 |
| RAB12 | 0.393517 | -0.13303 | -0.52654 | 4.70E-06 |
| RAB19 | -0.38485 | 0.11951 | 0.504356 | 0.000582 |
| RAB27B | -1.29903 | -0.22957 | 1.069463 | 6.41E-08 |
| RAB29 | 0.232257 | -0.13701 | -0.36926 | 0.002586 |
| RAB30 | -0.75316 | -0.16338 | 0.589785 | 0.000121 |
| RAB3A | -0.26816 | 0.041114 | 0.309278 | 0.007626 |
| RAB3D | -0.8655 | -0.25251 | 0.612987 | 0.000385 |
| RAB5B | -0.2059 | 0.024982 | 0.230881 | 0.008226 |
| RABEP1 | -0.75278 | -0.03951 | 0.713264 | 1.21E-09 |
| RABEP2 | -0.29187 | -0.00398 | 0.287888 | 7.23E-05 |
| RABEPK | -0.28366 | -0.02722 | 0.256445 | 0.00015 |
| RABGEF1 | -0.25363 | 0.193062 | 0.446689 | 1.34E-06 |
| RABGGTA | -0.04476 | 0.099661 | 0.144423 | 0.006378 |
| RABIF | 0.023551 | 0.337965 | 0.314414 | 0.000608 |
| RABL3 | -0.19863 | 0.063836 | 0.262463 | 0.000118 |
| RABL6 | -0.17779 | 0.081271 | 0.259058 | 0.000301 |
| RAD23B | -0.17023 | 0.02084 | 0.191066 | 0.008809 |
| RAD50 | -0.17992 | -0.01081 | 0.16911 | 0.004079 |
| RALGAPA1 | -0.22581 | 0.03156 | 0.257369 | 0.000621 |
| RALGAPB | -0.25636 | -0.01663 | 0.239729 | 0.0008 |
| RALGPS2 | -0.57067 | 0.154718 | 0.725385 | 1.68E-06 |
| RAP1GDS1 | -0.188 | 0.017343 | 0.205344 | 0.008377 |
| RAP2B | 0.430885 | 0.136714 | -0.29417 | 0.002838 |
| RAP2C | -0.51715 | -0.00786 | 0.509286 | 0.000241 |
| RAPGEF1 | 0.202537 | 0.02481 | -0.17773 | 0.007722 |
| RARA | -0.69853 | -0.08724 | 0.611283 | 5.75E-06 |
| RARRES1 | 0.368053 | -0.78503 | -1.15308 | 6.00E-05 |
| RASA1 | -0.17039 | 0.092953 | 0.263347 | 0.002655 |
| RASAL1 | 0.253807 | -0.63017 | -0.88398 | 0.00016 |
| RASEF | -0.57477 | -0.05726 | 0.517509 | 0.003815 |
| RASL12 | 0.406817 | 0.033365 | -0.37345 | 0.008026 |
| RASSF1 | 0.298659 | -0.01902 | -0.31768 | 0.007789 |
| RASSF5 | 0.438253 | 0.049275 | -0.38898 | 0.005457 |
| RAVER2 | 0.364995 | -0.01495 | -0.37994 | 0.000573 |
| RBKS | -0.61122 | 0.077802 | 0.689025 | 6.29E-07 |
| RBM27 | -0.10671 | 0.066851 | 0.173566 | 0.004209 |
| RBM3 | -0.4387 | -0.15315 | 0.285547 | 0.001016 |
| RBM34 | -0.03588 | -0.4048 | -0.36893 | 0.009664 |
| RBM47 | -0.42835 | 0.108263 | 0.536609 | 1.03E-06 |
| RBMS1 | 0.365529 | -0.02486 | -0.39039 | 0.000862 |
| RCC2 | 0.192321 | -0.09906 | -0.29138 | 0.002918 |
| RCN1 | 0.306986 | -0.04009 | -0.34708 | 0.001869 |
| RCOR2 | 0.537205 | 0.01337 | -0.52383 | 0.005168 |
| RCOR3 | -0.30435 | -0.05979 | 0.244558 | 0.005121 |
| RDH10 | 0.384654 | -0.3919 | -0.77656 | 1.87E-05 |
| RDX | 0.348268 | -0.07481 | -0.42308 | 0.000259 |
| REEP5 | -0.94292 | -0.47763 | 0.465285 | 0.001047 |
| REEP6 | -1.4181 | -0.64359 | 0.774512 | 1.29E-06 |
| RELB | 0.295315 | -0.05729 | -0.35261 | 0.000677 |
| REPS2 | -0.54776 | -0.084 | 0.463751 | 1.50E-05 |
| RERG | -0.33502 | 0.163557 | 0.498582 | 0.001575 |
| RETREG3 | -0.63846 | -0.25287 | 0.385591 | 0.001942 |
| REXO2 | 0.518937 | 0.117765 | -0.40117 | 0.000417 |
| RHEB | 0.289443 | -0.05283 | -0.34227 | 0.002223 |
| RHOB | -0.37647 | 0.046242 | 0.422708 | 0.000472 |
| RHOT1 | -0.05968 | 0.146269 | 0.205948 | 0.005503 |
| RHOT2 | -0.11084 | 0.154999 | 0.265842 | 0.009567 |
| RIC1 | 0.325742 | 0.051165 | -0.27458 | 0.007944 |
| RIC8B | -0.14312 | 0.070063 | 0.213183 | 0.009376 |
| RIF1 | 0.161245 | -0.21893 | -0.38018 | 0.00039 |
| RIN2 | -0.24997 | -0.00285 | 0.247121 | 0.006043 |
| RIN3 | 0.169551 | -0.04754 | -0.21709 | 0.007278 |
| RING1 | -0.10988 | 0.152377 | 0.262254 | 0.000146 |
| RIOK1 | 0.197204 | -0.18474 | -0.38195 | 0.000782 |
| RIOX1 | -0.12106 | 0.09271 | 0.213767 | 0.001084 |
| RIPK2 | 0.444687 | 0.142933 | -0.30175 | 0.005823 |
| RIT1 | 0.51501 | 0.1391 | -0.37591 | 0.000105 |
| RMDN3 | -0.25341 | 0.131111 | 0.384517 | 0.001816 |
| RMND1 | -1.07826 | -0.50313 | 0.575126 | 0.001689 |
| RNASEH1 | 0.329685 | -0.03159 | -0.36127 | 0.003597 |
| RNF219 | 0.30606 | -0.1223 | -0.42836 | 0.000193 |
| RNF31 | -0.0248 | 0.144146 | 0.168946 | 0.007544 |
| RNGTT | 0.374449 | 0.065074 | -0.30938 | 0.006046 |
| RNPEP | -0.33803 | 0.086881 | 0.424909 | 1.61E-06 |
| ROCK2 | 0.185566 | -0.09983 | -0.28539 | 0.003815 |
| RORA | 0.015796 | -0.1584 | -0.1742 | 0.009926 |
| RPIA | 0.340444 | 0.073259 | -0.26719 | 0.004038 |
| RPS12 | 0.089688 | -0.06611 | -0.1558 | 0.006038 |
| RPS2 | 0.111554 | -0.07408 | -0.18563 | 0.002251 |
| RPS27L | -0.21575 | 0.038371 | 0.254125 | 0.004587 |
| RPS6KA3 | 0.363554 | 0.082193 | -0.28136 | 0.001736 |
| RPS6KA5 | -0.30571 | 0.006771 | 0.312479 | 0.005731 |
| RPS6KB1 | -0.14762 | 0.100656 | 0.248275 | 0.001329 |
| RRM2B | -0.44328 | -0.01281 | 0.430477 | 0.00105 |
| RRP15 | 0.108352 | -0.20411 | -0.31247 | 0.007457 |
| RRP36 | 0.091005 | -0.25278 | -0.34379 | 0.008536 |
| RRP7A | 0.242945 | -0.02402 | -0.26697 | 0.000308 |
| RTKN | 0.151953 | -0.16108 | -0.31304 | 0.005754 |
| RUFY2 | -0.18775 | 0.070678 | 0.258426 | 0.000342 |
| RUNDC1 | -0.48224 | 0.128706 | 0.610943 | 7.97E-07 |
| RXRA | -0.35192 | 0.004198 | 0.356123 | 4.63E-05 |
| S100A1 | 0.97891 | -0.79429 | -1.7732 | 5.60E-06 |
| S100A3 | 0.655753 | 0.042051 | -0.6137 | 1.20E-05 |
| S100B | 0.967337 | -0.32422 | -1.29155 | 1.51E-05 |
| SAMD12 | -0.54863 | -0.14333 | 0.405297 | 0.007144 |
| SAR1B | -0.22407 | 0.134901 | 0.358973 | 0.001485 |
| SAV1 | 0.387888 | 0.026883 | -0.361 | 0.002981 |
| SCAMP1 | -0.65326 | -0.24722 | 0.406041 | 0.000294 |
| SCCPDH | -0.42063 | 0.166746 | 0.587376 | 0.000275 |
| SCFD1 | -0.09206 | 0.170777 | 0.262835 | 0.00142 |
| SCFD2 | 0.021787 | 0.20912 | 0.187332 | 0.003578 |
| SCGB1D2 | -1.36957 | -0.51286 | 0.856717 | 0.001879 |
| SCIN | -0.44911 | 0.047687 | 0.496799 | 0.000711 |
| SCMH1 | 0.445542 | 0.095893 | -0.34965 | 0.006305 |
| SCP2 | -0.28774 | -0.02101 | 0.266721 | 0.009129 |
| SCPEP1 | 0.292787 | -0.01068 | -0.30346 | 0.002209 |
| SCRN2 | -0.17004 | 0.171679 | 0.341718 | 0.007516 |
| SCRN3 | -0.165 | 0.062745 | 0.227746 | 0.005448 |
| SCUBE2 | -1.50079 | -0.70009 | 0.800706 | 0.002264 |
| SCYL3 | -0.17448 | 0.128472 | 0.302954 | 0.001471 |
| SDF2 | 0.32198 | 0.032824 | -0.28916 | 0.001477 |
| SDR16C5 | -1.2279 | -0.52672 | 0.70118 | 0.003722 |
| SDSL | -0.28122 | 0.064435 | 0.34565 | 0.003498 |
| SEC13 | -0.09181 | 0.110804 | 0.202613 | 0.001045 |
| SEC14L1 | -0.23139 | 0.067958 | 0.299346 | 0.00075 |
| SEC14L2 | -0.94543 | -0.14719 | 0.798234 | 6.36E-07 |
| SEC16A | -0.45208 | 0.044928 | 0.497012 | 2.79E-12 |
| SEC23B | -0.30542 | 0.072984 | 0.378405 | 0.000185 |
| SEC23IP | -0.21847 | 0.088174 | 0.30664 | 1.78E-06 |
| SEC24C | -0.10335 | 0.117229 | 0.220582 | 0.002656 |
| SEC24D | -0.09619 | 0.224458 | 0.320647 | 0.000737 |
| SEC31A | -0.08721 | 0.079351 | 0.16656 | 0.001827 |
| SEMA3C | -0.67407 | -0.12203 | 0.552041 | 1.82E-05 |
| SENP3 | 0.356668 | 0.077502 | -0.27917 | 0.00166 |
| SEPHS2 | -0.10121 | 0.247634 | 0.348845 | 0.000628 |
| SEPSECS | -0.17101 | 0.101668 | 0.272683 | 0.001987 |
| 8-Sep | -0.0834 | 0.210755 | 0.29416 | 0.002288 |
| SERPINB10 | -0.04165 | -0.69239 | -0.65074 | 0.003189 |
| SERPINB5 | 0.596198 | -0.83195 | -1.42815 | 9.07E-05 |
| SERPINB6 | -0.10242 | 0.295377 | 0.397793 | 0.002572 |
| SETD7 | -0.07511 | 0.224716 | 0.299828 | 0.002198 |
| SFRP1 | 1.069293 | -0.36564 | -1.43493 | 3.35E-10 |
| SFXN1 | -0.41567 | -0.02075 | 0.394916 | 2.35E-07 |
| SFXN2 | -0.68572 | 0.00427 | 0.689994 | 1.26E-05 |
| SFXN5 | -0.55567 | -0.04184 | 0.513828 | 6.25E-06 |
| SGSM3 | -0.61093 | 0.020814 | 0.631742 | 3.24E-05 |
| SH2B1 | 0.260944 | -0.02164 | -0.28259 | 0.001365 |
| SH3BGRL | -0.8825 | -0.14743 | 0.735076 | 4.92E-06 |
| SH3BP1 | 0.342449 | 0.045932 | -0.29652 | 0.000317 |
| SH3BP4 | -0.32069 | 0.103886 | 0.424578 | 0.001183 |
| SH3GLB2 | -0.23362 | 0.182848 | 0.416465 | 1.36E-05 |
| SHARPIN | -0.19463 | 0.006267 | 0.2009 | 0.002381 |
| SHPK | -0.17764 | 0.09013 | 0.267773 | 0.000931 |
| SHROOM1 | -0.32185 | 0.094791 | 0.416637 | 0.001802 |
| SHTN1 | -0.44812 | -0.05264 | 0.395478 | 0.000107 |
| SIDT2 | 0.255746 | -0.05148 | -0.30723 | 0.005093 |
| SIGIRR | -0.75569 | 0.031219 | 0.786911 | 4.61E-05 |
| SIK2 | 0.416592 | 0.0858 | -0.33079 | 0.005626 |
| SIRPA | 0.188642 | -0.29858 | -0.48723 | 0.001295 |
| SIRT3 | -0.18933 | 0.100614 | 0.289943 | 0.009675 |
| SKA1 | 0.549523 | 0.075199 | -0.47432 | 0.008725 |
| SKIV2L | -0.08197 | 0.121174 | 0.20314 | 0.000908 |
| SKP1 | -0.25086 | -0.08581 | 0.165046 | 0.001171 |
| SLC22A18 | -0.61452 | -0.21604 | 0.398484 | 0.009002 |
| SLC25A13 | 0.392185 | -0.01982 | -0.41201 | 0.001402 |
| SLC25A24 | -0.39254 | -0.04238 | 0.35016 | 0.004311 |
| SLC25A35 | -0.58854 | -0.15876 | 0.42978 | 0.005185 |
| SLC27A2 | -0.9704 | -0.28138 | 0.689021 | 5.50E-05 |
| SLC27A3 | -0.34105 | -0.01001 | 0.331042 | 0.000818 |
| SLC2A1 | 0.325178 | -0.43742 | -0.76259 | 1.27E-05 |
| SLC3A2 | 0.157222 | -0.40706 | -0.56428 | 0.003102 |
| SLC44A4 | -0.72921 | -0.08533 | 0.643877 | 0.002728 |
| SLC5A6 | 0.248665 | -0.25868 | -0.50734 | 0.005714 |
| SLC7A1 | 0.310671 | -0.21665 | -0.52732 | 0.00274 |
| SLC7A5 | -0.2383 | -1.04982 | -0.81152 | 0.002415 |
| SLC9A3R1 | -1.0801 | -0.21903 | 0.86107 | 9.42E-12 |
| SLC9A3R2 | -0.30953 | -0.01305 | 0.296485 | 0.001576 |
| SLFN11 | 0.344878 | 0.068342 | -0.27654 | 0.006303 |
| SLPI | -0.55243 | -1.49169 | -0.93926 | 0.006913 |
| SMARCC2 | -0.21827 | 0.053144 | 0.271409 | 2.14E-05 |
| SMC4 | 0.149415 | -0.19852 | -0.34793 | 0.003435 |
| SMCHD1 | 0.323036 | 0.017226 | -0.30581 | 0.000687 |
| SMOC1 | -0.11785 | -1.43745 | -1.3196 | 4.72E-05 |
| SND1 | 0.257132 | -0.00215 | -0.25928 | 0.000235 |
| SNF8 | -0.21656 | 0.086992 | 0.303557 | 1.07E-05 |
| SNIP1 | 0.246654 | -0.07024 | -0.31689 | 0.000258 |
| SNX24 | -0.23191 | 0.180229 | 0.412138 | 0.005069 |
| SNX27 | -0.02976 | 0.140011 | 0.169768 | 0.002165 |
| SOD1 | -0.25589 | 0.261195 | 0.517084 | 0.000931 |
| SORBS2 | 0.226561 | -0.37493 | -0.60149 | 0.002695 |
| SORCS2 | -0.17767 | 0.265824 | 0.443499 | 0.007502 |
| SORD | -0.79193 | -0.17107 | 0.620861 | 3.80E-05 |
| SOS1 | 0.247297 | -0.00223 | -0.24953 | 0.001964 |
| SOX10 | 0.91522 | -0.7632 | -1.67842 | 1.39E-06 |
| SOX9 | 0.174504 | -0.44928 | -0.62379 | 0.000373 |
| SPATA20 | -0.63039 | 0.009989 | 0.640381 | 1.07E-07 |
| SPDEF | -0.73275 | 0.03487 | 0.76762 | 4.05E-06 |
| SPG11 | -0.11057 | 0.081537 | 0.192109 | 0.004964 |
| SPHK1 | 0.363513 | -0.08782 | -0.45133 | 0.002586 |
| SPINT1 | 0.091712 | -0.29204 | -0.38376 | 0.007082 |
| SPR | -0.40689 | 0.174532 | 0.581422 | 2.12E-05 |
| SPTAN1 | 0.246574 | -0.04041 | -0.28699 | 0.000223 |
| SPTBN1 | 0.319075 | -0.00757 | -0.32664 | 0.000149 |
| SPTLC2 | -0.33874 | -0.01762 | 0.321122 | 0.001156 |
| SRA1 | -0.2027 | 0.123821 | 0.326518 | 6.52E-06 |
| SRCIN1 | -0.44599 | 0.04776 | 0.493755 | 0.000504 |
| SREK1 | -0.13989 | 0.101499 | 0.241385 | 0.003059 |
| SRF | 0.319468 | -0.24913 | -0.5686 | 1.92E-05 |
| SRP54 | -0.19369 | 0.032263 | 0.225951 | 5.09E-05 |
| SRP68 | -0.21542 | -0.02071 | 0.194706 | 0.000992 |
| SRPK1 | 0.135863 | -0.28539 | -0.42125 | 0.000156 |
| SRPRA | 0.179512 | -0.06012 | -0.23963 | 0.009249 |
| SRXN1 | -0.21874 | 0.127148 | 0.345889 | 0.001604 |
| SSBP1 | 0.415178 | 0.079001 | -0.33618 | 0.009755 |
| SSH3 | -0.54381 | 0.109033 | 0.652846 | 2.51E-10 |
| ST6GAL1 | 0.180709 | -0.23293 | -0.41364 | 0.000994 |
| STAC2 | -0.15654 | -1.19459 | -1.03805 | 0.00129 |
| STAM2 | -0.10022 | 0.234054 | 0.334272 | 7.24E-05 |
| STARD10 | -1.13182 | 0.089282 | 1.221102 | 7.82E-09 |
| STARD7 | 0.247313 | 0.001973 | -0.24534 | 0.000907 |
| STAT5A | 0.534974 | 0.172701 | -0.36227 | 0.00092 |
| STC2 | -1.0792 | -0.08116 | 0.998047 | 7.09E-07 |
| STEAP3 | 0.453128 | -0.30479 | -0.75792 | 8.94E-07 |
| STK11IP | -0.09779 | 0.17321 | 0.271005 | 0.001663 |
| STK16 | -0.16678 | 0.039577 | 0.206359 | 0.009015 |
| STK25 | -0.13039 | 0.075739 | 0.206133 | 0.003307 |
| STK3 | 0.433442 | 0.11127 | -0.32217 | 0.000621 |
| STK38 | 0.239098 | 0.00409 | -0.23501 | 0.002006 |
| STK39 | -0.42027 | 0.157026 | 0.577291 | 0.000192 |
| STMN1 | -0.04188 | -0.43493 | -0.39305 | 0.00886 |
| STOML2 | 0.022784 | -0.1986 | -0.22139 | 0.00516 |
| STON2 | -0.40485 | 0.004374 | 0.409222 | 0.001218 |
| STRN3 | -0.18548 | 0.13629 | 0.321775 | 6.08E-06 |
| STRN4 | -0.10932 | 0.03975 | 0.149069 | 0.003627 |
| STUB1 | -0.53244 | -0.03739 | 0.495048 | 7.19E-06 |
| STX12 | 0.255869 | -0.00866 | -0.26453 | 0.001729 |
| STXBP2 | -0.11972 | 0.103412 | 0.22313 | 0.002945 |
| STXBP4 | -0.42083 | -0.11732 | 0.303501 | 0.000384 |
| SUGP2 | -0.38694 | -0.09622 | 0.29072 | 0.000227 |
| SULT2B1 | -0.70576 | 0.082075 | 0.78784 | 2.50E-05 |
| SUOX | -0.39438 | 0.042297 | 0.436674 | 0.001094 |
| SUPT20H | 0.27234 | 0.00113 | -0.27121 | 0.00081 |
| SURF1 | -0.18772 | 0.155365 | 0.343087 | 0.001917 |
| SVIL | 0.2324 | -0.02405 | -0.25645 | 0.008589 |
| SYAP1 | -0.44401 | 0.083229 | 0.52724 | 1.17E-05 |
| SYBU | -0.54565 | 0.004962 | 0.550614 | 0.001642 |
| SYNCRIP | 0.307998 | 0.05187 | -0.25613 | 0.003238 |
| SYNM | 0.39196 | -0.60327 | -0.99523 | 2.70E-05 |
| SYNRG | -0.11349 | 0.067387 | 0.180876 | 0.001375 |
| SYT1 | -0.6964 | -0.12938 | 0.567022 | 0.000936 |
| SYT13 | -0.34202 | 0.024397 | 0.366421 | 0.005925 |
| SYTL1 | -0.44596 | -0.07849 | 0.367463 | 0.000985 |
| SYTL2 | -0.92651 | -0.14105 | 0.785454 | 5.68E-07 |
| SYTL4 | -0.72478 | 0.016631 | 0.741415 | 1.58E-09 |
| SYTL5 | -0.38493 | 0.126597 | 0.511531 | 0.001678 |
| TAB3 | -0.04239 | 0.177511 | 0.219905 | 0.0078 |
| TACC3 | 0.239682 | -0.0884 | -0.32808 | 0.009698 |
| TACO1 | -0.34109 | 0.007047 | 0.348134 | 0.000249 |
| TANC2 | -0.51844 | 0.09494 | 0.613378 | 9.90E-12 |
| TANK | 0.204908 | -0.21816 | -0.42307 | 0.001652 |
| TARBP1 | 0.309677 | -0.01653 | -0.32621 | 0.00039 |
| TBC1D17 | -0.14154 | 0.154092 | 0.29563 | 0.000427 |
| TBC1D30 | -0.54393 | -0.04926 | 0.494671 | 0.00112 |
| TBC1D4 | 0.170451 | -0.22166 | -0.39211 | 0.000168 |
| TBC1D9 | -0.73709 | 0.12712 | 0.864215 | 5.11E-09 |
| TBC1D9B | -0.19177 | 0.052224 | 0.243996 | 3.17E-05 |
| TBX3 | -0.73738 | -0.27287 | 0.464504 | 0.000168 |
| TC2N | -0.81428 | -0.09623 | 0.718051 | 8.04E-06 |
| TCEA3 | -0.58799 | -0.14758 | 0.440405 | 0.000497 |
| TCEAL1 | -0.83103 | -0.233 | 0.598033 | 3.26E-05 |
| TCEAL3 | -1.02374 | -0.52911 | 0.494632 | 0.001896 |
| TCEAL4 | -0.69978 | -0.26875 | 0.43103 | 0.004426 |
| TCF7L2 | 0.083729 | -0.35097 | -0.4347 | 0.00175 |
| TEAD3 | 0.287009 | -0.00086 | -0.28787 | 0.007712 |
| TES | 0.180685 | -0.26346 | -0.44414 | 0.000368 |
| TESK2 | -0.28654 | 0.115384 | 0.40192 | 9.73E-05 |
| TESMIN | -0.51562 | 0.014863 | 0.530479 | 0.002488 |
| TEX264 | -0.4133 | -0.15885 | 0.254449 | 0.00525 |
| TFAP2B | -0.60986 | -0.09476 | 0.515101 | 0.004331 |
| TFCP2L1 | 0.462091 | -0.07555 | -0.53764 | 0.009776 |
| TFF1 | -2.10483 | -1.3069 | 0.797934 | 0.000799 |
| TFF3 | -1.61069 | -0.25119 | 1.359502 | 8.80E-08 |
| TGFB3 | -0.52635 | -0.14994 | 0.376404 | 0.007092 |
| TGM2 | 0.235081 | -0.16791 | -0.40299 | 0.003935 |
| THAP4 | -0.36418 | 0.052485 | 0.416669 | 0.007837 |
| THBS4 | -0.53038 | 0.100693 | 0.631076 | 0.001527 |
| THEMIS2 | 0.536983 | 0.08822 | -0.44876 | 0.00058 |
| THNSL1 | 0.245597 | -0.2042 | -0.4498 | 0.000178 |
| THSD4 | -0.91859 | -0.12662 | 0.791964 | 4.06E-06 |
| THTPA | -0.22871 | 0.104226 | 0.332931 | 0.000276 |
| THUMPD3 | -0.13182 | 0.037074 | 0.168894 | 0.007508 |
| TIA1 | 0.28546 | -0.05564 | -0.3411 | 0.005981 |
| TIMM44 | 0.144407 | -0.15175 | -0.29616 | 0.001762 |
| TINAGL1 | 0.750849 | -0.03639 | -0.78724 | 3.03E-07 |
| TJAP1 | 0.207444 | -0.03785 | -0.24529 | 0.000384 |
| TJP3 | -0.61381 | -0.10289 | 0.51092 | 1.33E-06 |
| TKFC | -0.39472 | 0.21057 | 0.605288 | 1.75E-06 |
| TLDC1 | 0.35626 | -0.13503 | -0.49129 | 0.002513 |
| TLE1 | 0.117734 | -0.23145 | -0.34918 | 0.003576 |
| TLE3 | -0.47289 | 0.090858 | 0.563752 | 1.81E-09 |
| TLN1 | 0.242857 | 0.043758 | -0.1991 | 0.00804 |
| TM9SF1 | -0.75539 | -0.2133 | 0.542086 | 0.000767 |
| TMBIM6 | -0.37139 | 0.071567 | 0.442954 | 0.006198 |
| TMED7 | -0.05712 | 0.251537 | 0.308656 | 0.000309 |
| TMED8 | -0.07838 | 0.132824 | 0.211208 | 0.003313 |
| TMEM132A | 0.156742 | -0.15764 | -0.31439 | 0.004619 |
| TMEM141 | -0.21793 | 0.204663 | 0.422591 | 0.006902 |
| TMEM192 | -0.26293 | 0.133547 | 0.396478 | 0.004095 |
| TMEM205 | -0.96491 | -0.47293 | 0.491979 | 0.001971 |
| TMEM263 | -0.02335 | 0.282223 | 0.305575 | 0.007284 |
| TMEM63C | -0.32359 | 0.050817 | 0.37441 | 0.005542 |
| TMF1 | -0.36754 | -0.00063 | 0.366912 | 2.79E-09 |
| TMOD1 | 0.378067 | -0.05928 | -0.43735 | 0.001479 |
| TNFAIP3 | 0.337385 | -0.05737 | -0.39476 | 0.007762 |
| TOM1L1 | -0.71925 | 0.181363 | 0.900608 | 1.64E-06 |
| TOM1L2 | -0.22068 | 0.088397 | 0.309077 | 0.00715 |
| TP53BP1 | -0.26305 | 0.053378 | 0.316428 | 0.000321 |
| TP53BP2 | 0.379789 | -0.02812 | -0.40791 | 8.86E-07 |
| TP53RK | -0.16841 | 0.053992 | 0.222405 | 0.001244 |
| TPD52L2 | 0.199922 | -0.01979 | -0.21971 | 0.002195 |
| TPRG1 | -1.65161 | -0.78595 | 0.86566 | 0.004376 |
| TPRN | -0.27499 | 0.099961 | 0.37495 | 0.004483 |
| TPX2 | 0.269493 | -0.19741 | -0.4669 | 0.005852 |
| TRAF1 | 0.347939 | -0.05188 | -0.39982 | 0.002034 |
| TRAF2 | -0.24536 | 0.001937 | 0.247301 | 0.002235 |
| TRAF3IP1 | -0.46181 | -0.1083 | 0.353513 | 0.001148 |
| TRERF1 | -0.47769 | -0.05452 | 0.423171 | 0.002756 |
| TRIM2 | 0.698428 | -0.06835 | -0.76677 | 1.27E-07 |
| TRIM28 | -0.35074 | -0.05556 | 0.295183 | 0.004744 |
| TRIM29 | 0.426482 | -1.08046 | -1.50694 | 1.66E-06 |
| TRIM3 | -0.54348 | 0.102082 | 0.645559 | 1.82E-06 |
| TRIM32 | -0.03268 | 0.160523 | 0.193205 | 0.001037 |
| TRIM36 | -0.50334 | -0.12523 | 0.378109 | 0.009396 |
| TRIM47 | 0.324607 | 0.052013 | -0.27259 | 0.000789 |
| TRIO | -0.116 | 0.171564 | 0.287565 | 0.002985 |
| TRIP11 | -0.19562 | -0.05073 | 0.144891 | 0.005935 |
| TRIP6 | 0.217166 | -0.13034 | -0.34751 | 0.000561 |
| TRIT1 | 0.25359 | -0.06157 | -0.31516 | 0.008295 |
| TRMT6 | 0.176941 | -0.10305 | -0.27999 | 0.005632 |
| TRMT61A | 0.288374 | 0.02706 | -0.26131 | 0.000791 |
| TRMU | 0.245283 | -0.04374 | -0.28902 | 0.004059 |
| TSC22D3 | -0.3522 | 0.126643 | 0.478844 | 0.000102 |
| TSC22D4 | 0.344792 | 0.102826 | -0.24197 | 0.001264 |
| TSKU | -0.45335 | -0.13055 | 0.322808 | 0.006706 |
| TSPAN1 | -0.63356 | 0.183357 | 0.816921 | 0.00098 |
| TSPYL5 | 0.169754 | -0.87028 | -1.04003 | 1.00E-05 |
| TSTA3 | -0.30875 | 0.070489 | 0.379235 | 0.004185 |
| TSTD1 | -0.45028 | -0.08056 | 0.369713 | 0.008174 |
| TTC13 | 0.377424 | 0.047196 | -0.33023 | 1.02E-06 |
| TTC19 | -0.13769 | 0.082839 | 0.220534 | 0.003395 |
| TTC21B | -0.04204 | 0.185748 | 0.227787 | 0.000949 |
| TTC26 | -0.14528 | 0.149094 | 0.294379 | 0.000941 |
| TTC37 | -0.14544 | 0.04568 | 0.191117 | 0.001537 |
| TTC39A | -1.31654 | -0.4589 | 0.857647 | 1.48E-09 |
| TTC4 | 0.318102 | 0.106548 | -0.21155 | 0.0078 |
| TTC7A | 0.452782 | -0.02235 | -0.47513 | 7.29E-05 |
| TTC8 | -0.35187 | 0.091344 | 0.443218 | 1.71E-05 |
| TTK | 0.446876 | -0.08589 | -0.53276 | 0.001848 |
| TXNDC12 | 0.364007 | 0.022839 | -0.34117 | 0.000246 |
| TXNDC17 | -0.33922 | -0.01263 | 0.326594 | 0.003036 |
| TXNRD3 | 0.400818 | 0.065345 | -0.33547 | 0.00068 |
| TYMS | 0.33983 | -0.11613 | -0.45596 | 0.003442 |
| UAP1L1 | -0.0418 | 0.199522 | 0.241325 | 0.006519 |
| UBASH3B | 0.566885 | -0.11838 | -0.68526 | 1.52E-07 |
| UBE3A | -0.07766 | 0.086716 | 0.164374 | 0.003376 |
| UBR1 | -0.13926 | 0.095733 | 0.234991 | 3.10E-05 |
| UBTD2 | -0.26226 | 0.103124 | 0.365379 | 2.85E-06 |
| UBXN6 | -0.02178 | 0.236288 | 0.258067 | 0.003223 |
| UCHL1 | 0.379471 | -0.41526 | -0.79473 | 0.001038 |
| UEVLD | -0.09744 | 0.217372 | 0.314814 | 0.001403 |
| UFSP2 | -0.13638 | 0.222366 | 0.358742 | 0.001088 |
| UGDH | -1.17919 | -0.35752 | 0.821669 | 3.30E-06 |
| UGP2 | 0.432019 | -0.13228 | -0.5643 | 1.70E-07 |
| UNC119B | -0.08306 | 0.150798 | 0.233857 | 0.002604 |
| UNC5B | -0.02285 | 0.296968 | 0.319819 | 0.007875 |
| UPF2 | 0.313734 | 0.088738 | -0.225 | 0.001386 |
| UPF3B | 0.158469 | -0.13406 | -0.29253 | 0.000559 |
| UPP1 | 0.237954 | -0.09185 | -0.32981 | 0.005716 |
| UQCC1 | -0.35318 | 0.044352 | 0.39753 | 0.000494 |
| UQCC2 | -0.18163 | 0.298211 | 0.479838 | 0.000168 |
| UROS | -0.13703 | 0.079732 | 0.216759 | 0.008963 |
| USP1 | 0.118964 | -0.21412 | -0.33309 | 0.003855 |
| USP32 | -0.29393 | -0.01122 | 0.282704 | 0.004342 |
| USP37 | 0.117347 | -0.21064 | -0.32798 | 0.003228 |
| USP40 | -0.07206 | 0.189083 | 0.26114 | 0.003366 |
| USP47 | -0.05271 | 0.238251 | 0.290956 | 4.07E-06 |
| USP6NL | 0.24497 | -0.23026 | -0.47523 | 0.000388 |
| USP7 | -0.07921 | 0.115759 | 0.194971 | 0.008153 |
| USP8 | -0.14094 | 0.007537 | 0.148477 | 0.004385 |
| UTP4 | 0.228803 | -0.09051 | -0.31931 | 0.000384 |
| VAC14 | 0.235952 | 0.063852 | -0.1721 | 0.004155 |
| VASP | 0.191629 | -0.0661 | -0.25773 | 0.001201 |
| VAV2 | -0.19805 | 0.056753 | 0.254805 | 6.36E-05 |
| VAV3 | -0.39947 | 0.061425 | 0.4609 | 5.95E-05 |
| VDAC1 | -0.22342 | 0.151846 | 0.375262 | 0.000813 |
| VEZT | -0.45544 | -0.18421 | 0.271228 | 0.003046 |
| VGLL1 | 0.253143 | -0.15 | -0.40315 | 0.006098 |
| VLDLR | 0.314493 | -0.04825 | -0.36274 | 0.001511 |
| VPS13B | -0.14595 | 0.035945 | 0.181895 | 0.002893 |
| VPS37C | -0.36229 | 0.173258 | 0.535551 | 2.77E-05 |
| VPS41 | 0.015532 | 0.195212 | 0.179679 | 0.003695 |
| VTCN1 | 0.186126 | -0.80524 | -0.99136 | 0.001095 |
| VTN | 0.158439 | -0.22498 | -0.38342 | 0.005148 |
| VWA1 | 0.402341 | 0.097134 | -0.30521 | 0.002368 |
| VWA5A | -0.17201 | 0.174306 | 0.346314 | 0.005361 |
| WDHD1 | 0.23569 | -0.11143 | -0.34712 | 0.000253 |
| WDR19 | -0.20858 | 0.164528 | 0.373103 | 1.48E-05 |
| WDR24 | -0.12178 | 0.065571 | 0.18735 | 0.000878 |
| WDR35 | -0.09095 | 0.243051 | 0.333997 | 0.002422 |
| WDR44 | -0.0591 | 0.145934 | 0.205032 | 0.000687 |
| WDR54 | -0.40071 | 0.199837 | 0.600552 | 0.000308 |
| WDR55 | -0.04736 | 0.207931 | 0.255292 | 0.002489 |
| WDR74 | 0.26293 | -0.10215 | -0.36508 | 5.39E-05 |
| WFS1 | -0.72428 | -0.20999 | 0.514296 | 8.63E-06 |
| WIPF2 | -0.40767 | -0.13059 | 0.277082 | 0.006553 |
| WNK4 | -0.90258 | -0.24781 | 0.654774 | 3.09E-05 |
| WWP1 | -0.48912 | -0.12382 | 0.365306 | 0.005205 |
| WWTR1 | 0.330402 | -0.19783 | -0.52824 | 3.42E-05 |
| XPC | -0.28108 | 0.020256 | 0.301337 | 8.33E-05 |
| XPNPEP1 | 0.337604 | 0.065802 | -0.2718 | 0.000292 |
| XPO4 | 0.181462 | -0.01398 | -0.19544 | 0.009552 |
| XPO5 | 0.33778 | -0.20348 | -0.54126 | 0.0009 |
| XPO7 | 0.131252 | -0.10037 | -0.23163 | 0.008976 |
| XRN2 | -0.09102 | 0.075212 | 0.166232 | 0.003805 |
| YEATS2 | 0.2071 | -0.01718 | -0.22428 | 0.001839 |
| YES1 | 0.281052 | -0.06212 | -0.34317 | 0.000231 |
| YOD1 | 0.045722 | -0.3012 | -0.34692 | 0.008815 |
| YPEL2 | -0.25953 | 0.196079 | 0.455613 | 0.00569 |
| YTHDC2 | -0.07481 | 0.151542 | 0.226353 | 0.001783 |
| ZBTB7A | -0.12426 | 0.124176 | 0.248433 | 0.002071 |
| ZC3HC1 | 0.177566 | 0.015966 | -0.1616 | 0.004189 |
| ZCCHC11 | 0.334272 | 0.058786 | -0.27549 | 0.008927 |
| ZEB1 | -0.04655 | 0.272838 | 0.319393 | 0.006195 |
| ZFAND1 | 0.335193 | 0.023831 | -0.31136 | 0.00524 |
| ZFAND5 | 0.08163 | -0.25112 | -0.33275 | 0.002315 |
| ZFYVE1 | -0.13178 | 0.015773 | 0.14755 | 0.003754 |
| ZFYVE19 | -0.44234 | -0.05212 | 0.390221 | 6.01E-07 |
| ZMYND11 | 0.108635 | -0.17487 | -0.28351 | 0.006815 |
| ZNF121 | 0.468503 | 0.065395 | -0.40311 | 0.00032 |
| ZNF207 | -0.1134 | 0.071253 | 0.184657 | 0.004962 |
| ZNF264 | -0.37143 | 0.053143 | 0.424569 | 0.005481 |
| ZNF280C | 0.264128 | -0.21968 | -0.48381 | 0.000294 |
| ZNF346 | -0.1578 | 0.103521 | 0.261322 | 0.000112 |
| ZNF384 | 0.312382 | -0.02054 | -0.33292 | 0.002135 |
| ZNF385A | -0.75714 | -0.06708 | 0.690067 | 2.53E-10 |
| ZNF446 | -0.56809 | -0.1525 | 0.415593 | 0.000263 |
| ZNF462 | 0.516012 | -0.13424 | -0.65025 | 4.57E-06 |
| ZNF552 | -0.77641 | -0.23873 | 0.53768 | 2.89E-05 |
| ZNF609 | -0.45981 | -0.06015 | 0.399659 | 7.06E-05 |
| ZNF703 | -1.25612 | -0.55949 | 0.696625 | 0.000412 |
| ZNHIT2 | -0.17989 | 0.147299 | 0.327189 | 0.000101 |
| ZPR1 | 0.220878 | 0.045106 | -0.17577 | 0.005426 |
